# Postnatal gestational age determination by ultrasonographic measurement of transverse cerebellar diameter: A simulation study

**DOI:** 10.1371/journal.pone.0356370

**Published:** 2026-08-24

**Authors:** Andrea Pietravalle, Francesco Cavallin, Giovanni Putoto, Daniele Trevisanuto

**Affiliations:** 1 Doctors with Africa CUAMM, Padua, Italy; 2 Independent statistician, Solagna, Italy; 3 Department of Woman’s and Child’s Health, University of Padua, Padua, Italy; Aga Khan University - Kenya, KENYA

## Abstract

**Background:**

Accurate determination of gestational age (GA) has an important role in clinical practice, especially prevention/anticipation of prematurity-related complications. In low- and middle-income countries, the availability and accuracy of the gold standard for GA determination is very limited. In fact, prenatal ultrasonography in the first trimester is strongly affected by high costs, lack of skills and poor maternal access to health service. Furthermore, the determination by Last Menstrual Period has a very low accuracy, as well as postnatal clinical tools such as the Ballard Score. Recent studies in high-income countries tested TCD measurement by cranial ultrasound for GA determination with reported acceptable accuracy. The development of technology over the years is leading to a progressive spread of cranial ultrasound also in LMICs.

**Objective:**

To assess the performance of a published equation in estimating GA using fetal TCD measurements from low-income settings.

**Methods:**

Data were simulated using available information from relevant studies conducted in LMICs. Estimated GA was calculated as GA in weeks = 0.470 × TCD in mm + 13.162. The performance was assessed using modified calibration plots and Bland-Altman plots. Pooled bias and 95% limits of agreement (LoA) were also calculated.

**Results:**

Four datasets (n = 400, 257, 450, 500) were generated based on data from relevant studies. For GA 23–32 weeks, the equation was prone to overestimate and pooled bias was 6.0 days (95% LoA −6.7 to 18.7 days). For GA 33–36 weeks, the equation was prone to underestimate in one dataset and pooled bias was −1.5 days (95% LoA −16.8 to 13.8 days). For GA 37–40 weeks, the equation showed bad calibration and pooled bias was −5.0 days (95% LoA −25.8 to 15.8 days). When extending the original interval to GA 23−40 weeks, pooled bias was 2.8 days (95% LoA −13.0 to 18.6 days).

**Conclusions:**

Within the limitations of a simulation study, our findings suggest that the TCD-based equation may provide some benefits in postnatal estimation of GA between 23–32 weeks in low-resource settings. Lower precision, yet acceptable for clinical use, may be expected beyond 32 weeks’ gestation. Future studies may validate the equation in real newborns in low-resource settings where precise GA dating is available.

## 1. Introduction

Accurate determination of gestational age (GA) has an important role in clinical practice, allowing correct management of high-risk, complicated or post-date pregnancies and prevention/anticipation of prematurity-related complications [1]. The combination of an accurate last menstrual period (LMP) preceded by a normal cycle and an ultrasound measurement in the first trimester (up to 13 weeks + 6 day) is considered the gold standard for GA determination [1]. Ultrasound has an accuracy of ± 5–7 days in the first trimester, followed by ± 7–10 days in the second and ± 21–30 days in the third trimester [1]. In low- and middle-income countries (LMICs), the availability of prenatal ultrasonography is limited by high costs, lack of skills and poor maternal access to health service [2]. Furthermore, the determination by LMP has a very low accuracy of ± 3.7 weeks (26 days), as well as postnatal clinical tools such as the Ballard Score (BS) ± 3.7 weeks (26 days) [2,3].

These issues draw attention to finding alternative methods for accurate GA determination. Several studies suggested that fetal measurement of transverse cerebellar diameter (TCD) may be a good predictor of GA when compared to other clinical and fetal biometric parameters [4–14], even in the presence of abnormal skull shapes [4,15], fetal growth restriction [16,17], multiple pregnancies [18,19] and large-for-dates fetuses [5]. In a recent study from LMICs, a formula including TCD and femur length showed an accuracy of ultrasound of ± 10.5 days in the second and ± 15.1 days in the third trimester [20]. Davies et al. tested TCD measurement by cranial ultrasound for determining GA of preterm infants and defined an equation for prediction of GA (GA in weeks = 0.470 × TCD in mm + 13.162) with an accuracy of ± 16.3 days in the original study and ± 13.8 days in a subsequent study [21,22]. A recent study has strengthened the legitimacy of TCD measurement in the postnatal period, demonstrating a strong correlation between intrauterine and extrauterine ultrasound TCD measurement [23].

The development over the years of ultrasound technology into affordable, lightweight, portable devices has led to a progressive spread of the use of diagnostic methods such as brain ultrasound in newborns even in LMICs [24]. Although this would make it plausible to use cranial ultrasound to overcome the problem of obtaining accurate GA dating in low-income settings, the poor availability of reliable gold standard for GA dating makes it extremely difficult to replicate the study by Davies et al. [21] in low-income settings.

## 2. Aim

This study aimed to assess the performance of the equation by Davies at al. [21] in estimating GA using fetal TCD measurements from low-income settings.

## 3. Materials and methods

### 3.1. Study design

This is a simulation study evaluating the performance of the equation by Davies at al. [21] in estimating GA based on fetal TCD measurements. Data were simulated using available information from relevant studies conducted in LMICs.

### 3.2. Inclusion criteria for studies

The inclusion criteria were: studies conducted in LMICs; reporting of mean and standard deviation of fetal TCD measurements (mm) for each GA stratum (weeks); reliable assessment of GA based on gold standard (sure LMP or first-trimester ultrasound). There were no exclusion criteria. The relevant studies were identified via Pubmed search using the following key words: estimation, fetal biometry, gestational age, transverse cerebellar diameter, ultrasonography, developing countries. The search was carried out in January 2026 without time limitations.

### 3.3. Data collection

Information on study features (country, sample size, gestational age, GA assessment) and relevant data (mean and standard deviation of fetal TCD measurements for each week of GA) were retrieved from each study. Relevant data are reported in the S1 Table.

### 3.4. Statistical analysis

Four datasets were generated based on summary data from the relevant studies [25–28]. Each dataset included TCD and GA values, and had the same sample size of the corresponding source study. In each dataset, TCD values were simulated using means and standard deviations that were observed clinically and reported for each GA in weeks. Assuming Normal distribution for TCD, simulated data were generated using the *rnorm* function in R 4.5 (R Foundation for Statistical Computing, Vienna, Austria) [29]. The simulation process was checked using three repetitions for each dataset [30]: we aimed to obtain i) different simulated data in the three repetitions, and ii) simulated data consistent with the original data distribution. Estimated GA was calculated using the equation in Davies at al.: GA in weeks = 0.470 × TCD in mm + 13.162 [21]. The performance of the equation in estimating GA based on fetal TCD measurements was assessed using modified calibration plots and modified Bland-Altman plots. The modified calibration plot displayed mean estimated GA in y-axis and actual GA in x-axis; departures from the diagonal line indicated worse calibration. The modified Bland-Altman plot displayed the difference between estimated vs. actual GA in y-axis and actual GA in x-axis; bias (i.e., mean difference) and 95% limits of agreement (LoA) were plotted as horizontal lines. Pooled bias and 95% LoA were calculated according to the framework by Tipton et al. using a random-effects model [31]. Pooled bias was calculated as ∑ w_1j_D_j_/∑ w_1j_, where D_j_ is the bias in study j and the weights w_1j_ are defined to be inverse-variance. LoA were calculated as D ± 2√{exp[log(S^2^)]+T^2^}, where S^2^ is the estimated average study-variation and T^2^ is the between-study heterogeneity in bias. The main analysis was restricted to GA within 23–32 weeks, as the equation was developed in such domain [21]. The secondary analyses evaluated the performance of the equation also in different strata (GA 33–36 weeks and 37–40 weeks) to explore its applicability beyond the original domain. Pooled bias was also calculated for GA 23–36 and 23–40 weeks. In addition, a classification analysis for clinically relevant thresholds (extreme preterm, very preterm, late preterm, term) was performed by calculating positive and negative likelihood ratios using the package *mada* [32]. These metrics were preferred to sensitivity and specificity because these are interrelated hence pooling can be misleading, and the bivariate approach could not be employed due to the small number of studies [33,34]. A sensitivity analysis simulated data of TCD assuming different weekly distributions (uniform, skewed, heavy tail) and used these data to estimate GA according to the equation by Davies et al. [21]. Pooled bias and 95% LoA were calculated for each assumption. All analyses were carried out using R 4.5 (R Foundation for Statistical Computing, Vienna, Austria) [29]. The R code is available as an online supplement.

## 4. Results

### 4.1. Summary of the studies used for the simulation process

Four studies met the inclusion criteria and were used for the simulation process (S1 Fig) [25–28]. The main features of the studies are summarized in Table 1. The study #1 measured the TCD in 400 pregnant women with a singleton fetus (GA 14–38 weeks) and assessed the GA according to the LMP validated with early first trimester ultrasound (<10 weeks) [25]. The study #2 measured the TCD in 257 pregnant women with a singleton fetus (GA 16–40 weeks) and calculated the GA from the sure date of LMP onset [26]. The study #3 measured the TCD in 450 pregnant women with a singleton fetus (GA 13–42 weeks) and calculated the GA from the sure date of LMP onset [27]. The study #4 measured the TCD in 500 pregnant women with a singleton fetus (GA 18–40 weeks) and calculated the GA from the sure date of LMP onset [28]. All four studies employed a basic standard methodological approach to evaluate the TCD during routine prenatal fetal ultrasound (S2 Table).

**Table 1:** Summary of the included studies. GA: gestational age. LMP: last menstrual period. TCD: transverse cerebellar diameter.

Overall Information	Study#1	Study#2	Study#3	Study#4
First author	Adelabu	Eze	Adeyekun	Kummari
Country	Nigeria	Nigeria	Nigeria	India
Sample size	400	257	450	500
Gestational age	14-38 weeks	16-40 weeks	13-42 weeks	18-40 weeks
Assessment of GA	LMP validated with early first trimester ultrasound (<10 weeks)	Sure date of LMP onset	Sure date of LMP onset	Sure date of LMP onset

### 4.2. Simulated data

The simulation process was checked using three repetitions for each dataset, which correctly produced different simulated data (S2-S5 Figs) that were consistent with the original data distribution (S3-S6 Tables). The simulated datasets used in the analysis are reported as Supplementary File.

### 4.3. Analysis of the simulated data (GA 23–32 weeks)

**Fig 1:**
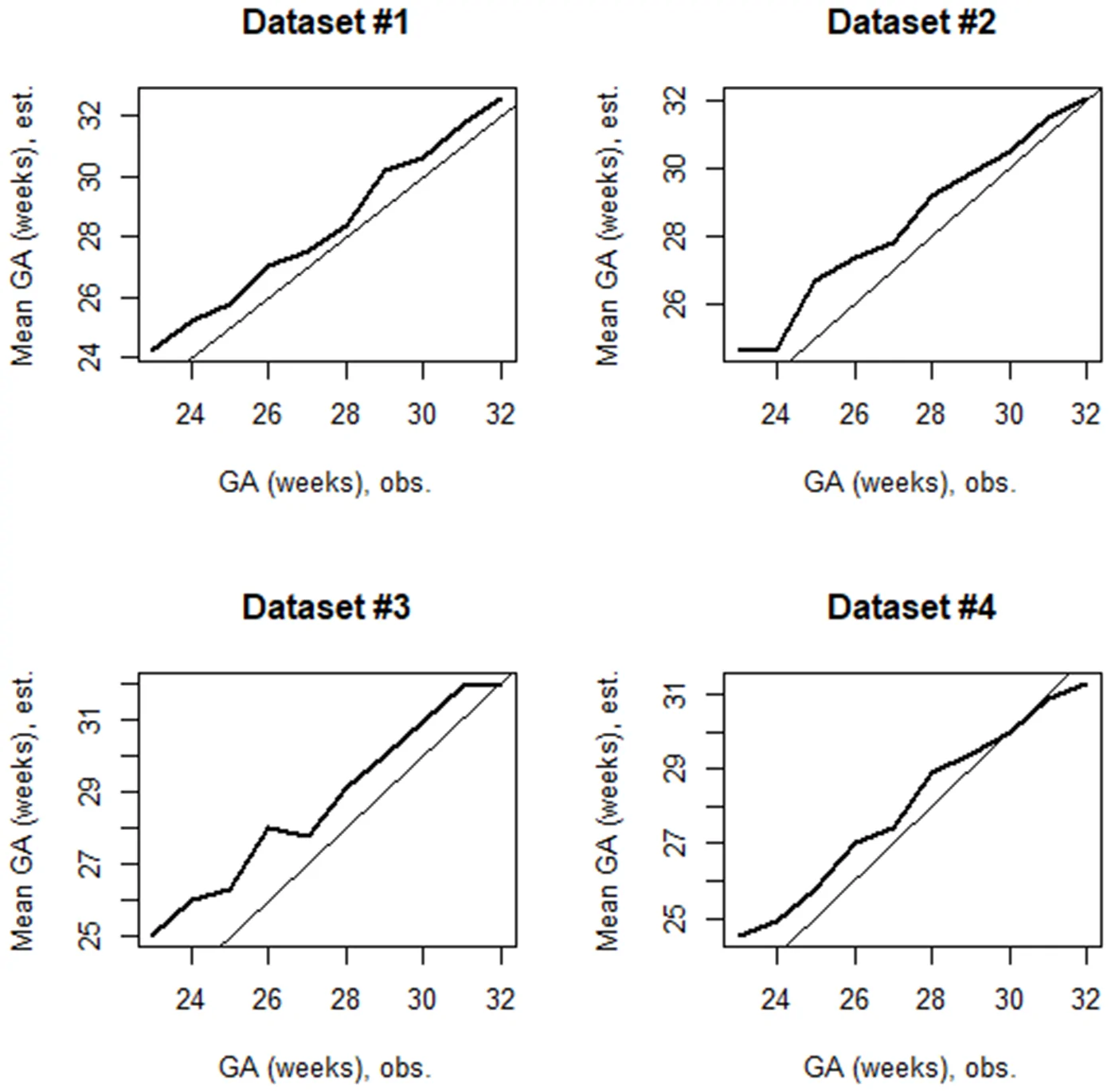
Modified calibration plot for GA within 23-32 weeks: mean estimated gestational age (est.) vs. actual gestational age (obs. ) for each simulated dataset. Departures from the diagonal line indicate worse calibration. GA: gestational age.

**Fig 2:**
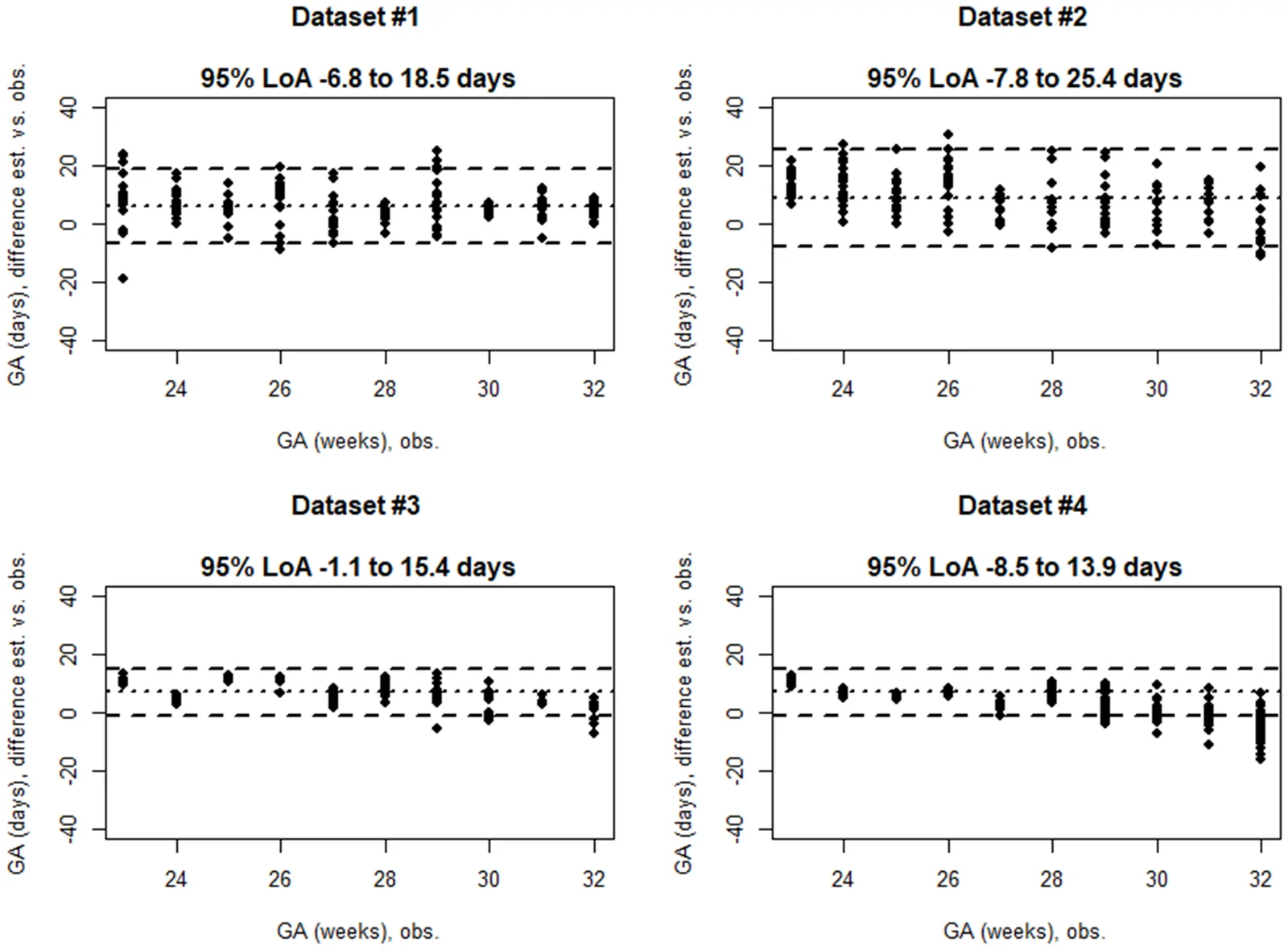
Modified Bland-Altman plot for GA within 23-32 weeks: the y-axis shows the difference between estimated GA (est.) vs. actual GA (obs. ); the x-axis shows the actual GA (obs.). GA: gestational age. LoA: limits of agreement.

For GA within 23–32 weeks, the equation was prone to overestimate actual GA in all datasets (Fig 1). GA mean difference was 5.9 days (95% LoA −6.8 to 18.5 days) in dataset #1, 8.8 days (95% LoA −7.8 to 25.4 days) in dataset #2, 7.1 days (95% LoA −1.1 to 15.4 days) in dataset #3, and 2.7 days (95% LoA −8.5 to 13.9 days) in dataset #4 (Fig 2). Pooled bias was 6.0 days (95% LoA −6.7 to 18.7 days) with very small heterogeneity (τ2 = 0).

### 4.4. Analysis of the simulated data (GA 33–36 weeks)

For GA within 33–36 weeks, the equation was prone to underestimate actual GA in one dataset while there was not a clear tendency in the others (S6 Fig). GA mean difference was 0.6 days (95% LoA −17.0 to 18.1 days) in dataset #1, 1.1 days (95% LoA −16.3 to 18.5 days) in dataset #2, 0.5 days (95% LoA −9.4 to 10.5 days) in dataset #3, and −7.3 days (95% LoA −18.7 to 4.2 days) in dataset #4 (S7 Fig). Pooled bias was −1.5 days (95% LoA −16.8 to 13.8 days) with small heterogeneity (τ2 = 0.16). When extending the original interval to GA 23−36 weeks, pooled bias was 4.2 days (95% LoA −9.8 to 18.1 days) with very small heterogeneity (τ2 = 0).

### 4.5. Analysis of the simulated data (GA 37–40 weeks)

For GA within 37–40 weeks, the equation showed bad calibration in datasets #2-3-4 (S8 Fig). GA mean difference was 0.6 days (95% LoA −16.3 to 17.5 days) in dataset #2, −0.7 days (95% LoA −13.0 to 11.7 days) in dataset #3, and −14.7 days (95% LoA −28.2 to −1.2 days) in dataset #4 (S9 Fig). Pooled bias was −5.0 days (95% LoA −25.8 to 15.8 days) with large heterogeneity (τ2 = 1.28). Dataset #1 could not be used due to the lack of TCD measurements for GA in 39−40 weeks. When extending the original interval to GA 23−40 weeks, pooled bias was 2.8 days (95% LoA −13.0 to 18.6 days) with small heterogeneity (τ2 = 0.17).

### 4.6. Classification analysis for clinically relevant thresholds

Table 2 shows the classification analyses for clinically relevant thresholds (extreme preterm, very preterm, late preterm, term). The positive and negative likelihood ratios suggested that classification based on equation-estimated GA had high discriminatory ability, although the performance decreased from extreme preterm to term thresholds.

**Table 2:** Classification analyses for clinically relevant thresholds.

Clinically relevant thresholds	Metrics	Estimate (95% confidence interval)
Extreme preterm	Positive likelihood ratio	96.79 (43.67 to 214.52)
	Negative likelihood ratio	0.04 (0.02 to 0.08)
Very preterm	Positive likelihood ratio	20.01 (12.99 to 30.83)
	Negative likelihood ratio	0.24 (0.12 to 0.47)
Late preterm	Positive likelihood ratio	15.95 (7.58 to 33.59)
	Negative likelihood ratio	0.34 (0.26 to 0.44)
Term	Positive likelihood ratio	8.82 (6.12 to 12.71)
	Negative likelihood ratio	0.15 (0.04 to 0.49)

### 4.7. Sensitivity analysis

The sensitivity analysis suggested comparable GA estimates when different weekly distributions (Normal, uniform, skewed, heavy tail) were used to simulate TCD data. For GA within 23–32 weeks, pooled bias was 6.0 days (95% LoA −6.7 to 18.7 days) with Normal distribution, 6.1 days (95% LoA −6.4 to 18.8 days) with uniform distribution, 6.2 days (95% LoA −6.3 to 18.9 days) with skewed distribution, and 5.9 days (95% LoA −7.1 to 18.9 days) with heavy-tailed distribution. For GA within 23–40 weeks, pooled bias was 2.8 days (95% LoA −13.0 to 18.6 days) with Normal distribution, 2.6 days (95% LoA −13.5 to 18.6 days) with uniform distribution, 2.9 days (95% LoA −13.4 to 19.1 days) with skewed distribution, and 2.6 days (95% LoA −13.9 to 19.1 days) with heavy-tailed distribution. All numerical results are reported in S7 Table.

## 5. Discussion

In resource-limited settings, several barriers hinder access to prenatal care, and obtaining accurate GA dating based on the gold standard (sure LMP or first-trimester ultrasound) is difficult [35]. In fact, antenatal visits take place mostly at Primary Health Care centers, mobile clinics and village health posts, where access to expensive equipment such as an ultrasound scanner and necessary skills are unlikely. On the contrary, the ever-increasing diffusion of portable and reliable ultrasound machines at hospital level and their growing use in neonatal brain ultrasound makes it plausible to use this tool to overcome the difficulty of obtaining accurate GA dating [24]. Davies et al. proposed an equation based on TCD measurement by cranial ultrasound for determining GA of preterm infants [21].

Our simulation-based evaluation suggested that such equation may provide some benefits in estimating GA between 23–32 weeks, which was the original domain of the equation [21]. When the equation was extended beyond the original domain, the between-study heterogeneity increased and the agreement slightly impaired as could be expected. These findings are correlated as higher between-study heterogeneity produces larger agreement intervals [31], and suggests caution in using the equation beyond the original domain. The agreement intervals indicated a magnitude of disagreement between estimated GA and actual GA between −1.4 and +2.6 weeks in GA 23–36 weeks, and between −1.8 and 2.7 weeks in GA 23–40 weeks. A disagreement of approximately ±2 weeks can be considered acceptable and may provide clinically useful support for broad gestational-age categorization in low-resource settings where reliable prenatal dating is unavailable. Specifically, it may reduce the risk of failing to identify extremely preterm (<28 gestational weeks) and very preterm (28–31 gestational weeks) infants. Accuracy may be lower for moderate preterm (32–33 gestational weeks) and late preterm (34–36 gestational weeks), but the clinical risk is also progressively reduced for these age groups. Nonetheless, the classification in clinically relevant strata (extreme preterm, very preterm, late preterm, term) based on equation-estimated GA suggested high discriminatory ability, suggesting an overall clinical utility. Of note, the performance decreased from extreme preterm to term strata, which may be due to the original domain of the equation [21].

The literature suggests that the New Ballard Score may overestimate GA by 3 days, with a 95% confidence interval of ±2.57 weeks (24 days) in high-resource settings [36]. This is comparable to the performance of the original Ballard Score, which may predict GA with a 95% confidence interval of ±2.86 weeks (20 days) for infants in 20–44 weeks’ gestation, and ±3.10 weeks (22 days) for infants <26 weeks’ gestation [36]. Other measurements supported the inaccuracy of the New Ballard Score in infants younger than 28 weeks’ gestation [37]. In low-resource settings, the accuracy of the Ballard Score and LMP are estimated to be ± 3.7 weeks (26 days) [2,3].

When indirectly compared to the performance of these approaches, we believe that the equation by Davies at al. may find a potential clinical application in low-resource settings. Of note, we calculated the 95% LoA (showing the interval where 95% of the differences between two measurements are expected to lie) while previous studies reported the 95% confidence interval of the difference (showing the interval where we expect the true difference to fall between if we repeat the study multiple times) [38,39]. Nevertheless, we think that the scale of disagreement between equation-estimated GA and actual GA would not clinically influence the management of most infants, hence providing a useful tool in low-resource settings. However, the findings in different GA strata (23–32, 33–36, and 37–40 weeks) suggest that the user should tailor the level of accuracy and interpretation according to the specific GA interval, ensuring more context-aware clinical use.

It should be underlined that Davies’s equation was originally developed using postnatal ultrasound, while all source measurements were prenatal fetal measurements [21,25–28]. The equation aimed to refine the use of TCD as reliable predictor of GA, and recent evidence demonstrated strong agreement between prenatal and postnatal TCD measurements [23], but prospective validation using real postnatal measurements remains necessary.

We acknowledge that the accuracy of a postnatal TCD evaluation depends on the operator’s skill, which may require targeted training programs and ongoing technical support to deploy its full potential in practice. However, we may speculate that ultrasound measurement following adequate training may be more accurate than clinical assessment using tools such as the Ballard Score. In this regard, further studies in low-resource settings might compare non-expert operators with expert operators in using the Ballard Score or measuring TCD.

The main limitation of our study is the use of algorithm-generated values to assess the performance of Davies’s equation in estimating GA using fetal TCD measurements [21]. Simulated data may oversimplify real-world data, hence failing to replicate patient heterogeneity. In this perspective, our findings provide promising information on the potential application of the equation, but clinical validation remains a paramount to assess its utility in the real world. In addition, the true distribution of TCD may be non-Normal, but the sensitivity analysis suggested comparable GA estimates when different weekly distributions (Normal, uniform, skewed, heavy tail) were used to simulate TCD data. Finally, the quality of the simulation depends on the quality of both assumptions and input data, hence suggesting caution in the interpretation of the results. Unfortunately, original raw data were not provided in the papers, hence we could not use raw data to inform the simulation process or quantify the bias that arose from our simulation process.

Overall, the generalizability of the findings may be limited to LMICs due to the inclusion criteria, while geographic and phenotypic representativeness was limited by the literature output that yielded only three studies from Nigeria and one study from India. Population-specific differences in fetal anthropometry and growth patterns could theoretically affect the performance of a gestational-age prediction model when applied across different settings. Indeed, ethnic differences in TCD growth curves have been reported, suggesting that population-specific reference charts may improve accuracy in some settings [40,41]. However, the original postnatal TCD equation was developed in Australia and subsequently evaluated in an independent cohort from Spain, showing comparable performance despite the different geographic and population settings [21,22]. Nevertheless, some degree of population-specific variability cannot be excluded, thus caution is suggested in the interpretation of our findings that may be integrated by future research using individual-level data from geographically different populations. Moreover, we cannot exclude some heterogeneity in TCD measurement technique across the studies, despite the employment of a basic standard methodological approach to evaluate the TCD during routine prenatal fetal ultrasound [25–28]. Such heterogeneity may be due to minor differences in image acquisition, caliper placement, transducer orientation and operator expertise. Since Davies’s equation estimates GA using TCD measures [21], these methodological differences may proportionally translate into slightly different GA estimates and contribute to between-study heterogeneity, which is accounted for by the random-effects pooling of the bias.

Due to the key limitation of using simulated data (although derived from actual fetal measurements), the true utility of this tool as clinical support will only be known after validation in real clinical settings and future studies may validate the equation by Davies et al. [21] using TCD measurements from real newborns in low-resource settings where precise GA dating is available. The inherent condition for real-world implementation is clearly the availability of an ultrasound. Despite recent developments of such technology have led to affordable devices and progressive spread in the diagnostics process, local constraints such as limited access to ultrasound equipment and limited expertise can impair the implementation of Davies’s equation in the clinical environments of some LMICs.

## 6. Conclusions

The use of postnatal TCD measurement through cranial ultrasonography appears to be a promising tool for determining GA in low-resource settings where the gold standard is difficult to apply. Further studies are needed to confirm and validate this finding.

## Supporting information

S1 Table Relevant data extracted from the selected studies. GA: gestational age. NA: not available. SD: standard deviation. TCD: transverse cerebellar diameter.
(DOCX)

S2 Table Description of the technique used in each source study to measure the transverse cerebellar diameter (TCD). (DOCX)

S3 Table The simulation process was checked using three repetitions for Study #1, which correctly produced consistent simulated data. GA: gestational age. SD: standard deviation. TCD: transverse cerebellar diameter.
(DOCX)

S4 Table The simulation process was checked using three repetitions for Study #2, which correctly produced consistent simulated data. GA: gestational age. SD: standard deviation. TCD: transverse cerebellar diameter.
(DOCX)

S5 Table The simulation process was checked using three repetitions for Study #3, which correctly produced consistent simulated data. GA: gestational age. SD: standard deviation. TCD: transverse cerebellar diameter.
(DOCX)

S6 Table The simulation process was checked using three repetitions for Study #4, which correctly produced consistent simulated data. GA: gestational age. SD: standard deviation. TCD: transverse cerebellar diameter.
(DOCX)

S7 Table Sensitivity analyses: simulated data of transverse cerebellar diameter were obtained assuming different weekly distributions (Normal, uniform, skewed, heavy tail) and used to estimate gestational age according to the equation by Davies et al. The pooled bias (difference between estimated vs. actual gestational age) and 95% limits of agreement suggested comparable estimates assuming different weekly distribution. (DOCX)

S1 Fig PRISMA diagram of included studies. (TIF)

S2 Fig The simulation process was checked using three repetitions for Study #1, which correctly produced different simulated data. Each set of pairs of simulated data is colored differently.Figure S3. The simulation process was checked using three repetitions for Study #2, which correctly produced different simulated data. Each set of pairs of simulated data is colored differently.
(TIF)

S4 Fig The simulation process was checked using three repetitions for Study #3, which correctly produced different simulated data. Each set of pairs of simulated data is colored differently.
(TIF)

S5 Fig The simulation process was checked using three repetitions for Study #4, which correctly produced different simulated data. Each set of pairs of simulated data is colored differently.
(TIF)

S6 Fig Modified calibration plot for GA within 33–36 weeks: mean estimated gestational age (est.) vs. actual gestational age (obs. ) for each simulated dataset. Departures from the diagonal line indicate worse calibration. GA: gestational age.
(TIF)

S7 Fig Modified Bland-Altman plot for GA within 33–36 weeks: the y-axis shows the difference between estimated GA (est.) vs. actual GA (obs. ); the x-axis shows the actual GA (obs.). GA: gestational age. LoA: limits of agreement.
(TIF)

S8 Fig Modified calibration plot for GA within 37–40 weeks: mean estimated gestational age (est.) vs. actual gestational age (obs. ) for each simulated dataset. Departures from the diagonal line indicate worse calibration. Dataset #1 could not be used due to the lack of TCD measurements for GA in 39–40 weeks. GA: gestational age.
(TIF)

S9 Fig Modified Bland-Altman plot for GA within 37–40 weeks: the y-axis shows the difference between estimated GA (est.) vs. actual GA (obs.); the x-axis shows the actual GA (obs.). Dataset #1 could not be used due to the lack of TCD measurements for GA in 39–40 weeks. GA: gestational age. LoA: limits of agreement.
(TIF)

S1 File Supplementary Material - R code. (PDF)

## Abbreviations

GA: Gestational Age
LMP: Last Menstrual Period
LMICs: Low- and Middle-Income Countries
TCD: Transverse Cerebellar Diameter
BS: Ballard Score
NBS: New Ballard Score
