# Supplementary material for: Postnatal gestational age determination by ultrasonographic measurement of transverse cerebellar diameter: A simulation study
Source: PLoS One. 2026 Aug 24;21(8):e0356370. doi: 10.1371/journal.pone.0356370 (PMC13502679; doi:10.1371/journal.pone.0356370)
Supplement: Supplementary file 1 [file pone.0356370.s016.pdf]

```
rm(list=ls(all=TRUE))


################ IMPORT:

#import the following information for each study:
#
#Menstrual_GA_weeks
#TCD_mm_mean
#TCD_mm_sd
#n_obs

library("readxl")

imp1 = read_excel("PATH\\data.xlsx", sheet="nigeria1_tab1")
imp2 = read_excel("PATH\\data.xlsx", sheet="nigeria2_tab1")
imp3 = read_excel("PATH\\data.xlsx", sheet="nigeria3_tab1")
imp4 = read_excel("PATH\\data.xlsx", sheet="india_tab1")

imp1<-data.frame(imp1)
imp2<-data.frame(imp2)
imp3<-data.frame(imp3)
imp4<-data.frame(imp4)

#domains:
#DAVIES 23-32 wk
#nig1 14-38 wk
#nig2 13-42 wk
#nig3 16-40 wk
#ind 18-40 wk
```

```
################ SIMULATION (NORMAL DISTRIBUTION):

set.seed(2179)

#niter is not used in rnorm function

xx<-imp1
      GAwk=rep(NA,1)
      TCDmm=rep(NA,1)
      cumul<-cumsum(xx$n_obs)
      for (i in 1:dim(xx)[1]) {
      GAwk<-c( GAwk,rep(xx$Menstrual_GA_weeks[i],xx$n_obs[i]) )
      TCDmm<-c(TCDmm,
round(rnorm(n=xx$n_obs[i],mean=xx$TCD_mm_mean[i],sd=xx$TCD_mm_sd[i]),1) )}
nig1<-data.frame(GAwk=GAwk[2:length(GAwk)],TCDmm=TCDmm[2:length(TCDmm)])

xx<-imp2
      GAwk=rep(NA,1)
      TCDmm=rep(NA,1)
      cumul<-cumsum(xx$n_obs)
      for (i in 1:dim(xx)[1]) {
      GAwk<-c( GAwk,rep(xx$Menstrual_GA_weeks[i],xx$n_obs[i]) )
      TCDmm<-c(TCDmm,
round(rnorm(n=xx$n_obs[i],mean=xx$TCD_mm_mean[i],sd=xx$TCD_mm_sd[i]),1) )}
nig2<-data.frame(GAwk=GAwk[2:length(GAwk)],TCDmm=TCDmm[2:length(TCDmm)])

xx<-imp3
      GAwk=rep(NA,1)
      TCDmm=rep(NA,1)
      cumul<-cumsum(xx$n_obs)
      for (i in 1:dim(xx)[1]) {
      GAwk<-c( GAwk,rep(xx$Menstrual_GA_weeks[i],xx$n_obs[i]) )
      TCDmm<-c(TCDmm,
round(rnorm(n=xx$n_obs[i],mean=xx$TCD_mm_mean[i],sd=xx$TCD_mm_sd[i]),1) )}
nig3<-data.frame(GAwk=GAwk[2:length(GAwk)],TCDmm=TCDmm[2:length(TCDmm)])

xx<-imp4
      GAwk=rep(NA,1)
      TCDmm=rep(NA,1)
      cumul<-cumsum(xx$n_obs)
      for (i in 1:dim(xx)[1]) {
      GAwk<-c( GAwk,rep(xx$Menstrual_GA_weeks[i],xx$n_obs[i]) )
      TCDmm<-c(TCDmm,
round(rnorm(n=xx$n_obs[i],mean=xx$TCD_mm_mean[i],sd=xx$TCD_mm_sd[i]),1) )}
ind<-data.frame(GAwk=GAwk[2:length(GAwk)],TCDmm=TCDmm[2:length(TCDmm)])

nig1$id<-"nig1"
nig2$id<-"nig2"
nig3$id<-"nig3"
ind$id<-"ind"

library("writexl")

write_xlsx(data.frame(rbind(nig1,nig2,nig3,ind)),"PATH\\simulated_data.xlsx")
```

```
################ CHECK SIMULATION PROCESS (n=3):

set.seed(2179)

#niter is not used in rnorm function


### CREATE:

xx<-imp1

      GAwk=rep(NA,1)
      TCDmm=rep(NA,1)
      cumul<-cumsum(xx$n_obs)

      for (i in 1:dim(xx)[1]) {
      GAwk<-c( GAwk,rep(xx$Menstrual_GA_weeks[i],xx$n_obs[i]) )
      TCDmm<-c(TCDmm,
round(rnorm(n=xx$n_obs[i],mean=xx$TCD_mm_mean[i],sd=xx$TCD_mm_sd[i]),1) )}

nig1a<-data.frame(GAwk=GAwk[2:length(GAwk)],TCDmm=TCDmm[2:length(TCDmm)])

      GAwk=rep(NA,1)
      TCDmm=rep(NA,1)
      cumul<-cumsum(xx$n_obs)

      for (i in 1:dim(xx)[1]) {
      GAwk<-c( GAwk,rep(xx$Menstrual_GA_weeks[i],xx$n_obs[i]) )
      TCDmm<-c(TCDmm,
round(rnorm(n=xx$n_obs[i],mean=xx$TCD_mm_mean[i],sd=xx$TCD_mm_sd[i]),1) )}

nig1b<-data.frame(GAwk=GAwk[2:length(GAwk)],TCDmm=TCDmm[2:length(TCDmm)])

      GAwk=rep(NA,1)
      TCDmm=rep(NA,1)
      cumul<-cumsum(xx$n_obs)

      for (i in 1:dim(xx)[1]) {
      GAwk<-c( GAwk,rep(xx$Menstrual_GA_weeks[i],xx$n_obs[i]) )
      TCDmm<-c(TCDmm,
round(rnorm(n=xx$n_obs[i],mean=xx$TCD_mm_mean[i],sd=xx$TCD_mm_sd[i]),1) )}

nig1c<-data.frame(GAwk=GAwk[2:length(GAwk)],TCDmm=TCDmm[2:length(TCDmm)])



xx<-imp2

      GAwk=rep(NA,1)
      TCDmm=rep(NA,1)
      cumul<-cumsum(xx$n_obs)

      for (i in 1:dim(xx)[1]) {
      GAwk<-c( GAwk,rep(xx$Menstrual_GA_weeks[i],xx$n_obs[i]) )
      TCDmm<-c(TCDmm,
round(rnorm(n=xx$n_obs[i],mean=xx$TCD_mm_mean[i],sd=xx$TCD_mm_sd[i]),1) )}

nig2a<-data.frame(GAwk=GAwk[2:length(GAwk)],TCDmm=TCDmm[2:length(TCDmm)])

      GAwk=rep(NA,1)
      TCDmm=rep(NA,1)
      cumul<-cumsum(xx$n_obs)

      for (i in 1:dim(xx)[1]) {
      GAwk<-c( GAwk,rep(xx$Menstrual_GA_weeks[i],xx$n_obs[i]) )
```

```
      TCDmm<-c(TCDmm,
round(rnorm(n=xx$n_obs[i],mean=xx$TCD_mm_mean[i],sd=xx$TCD_mm_sd[i]),1) )}

nig2b<-data.frame(GAwk=GAwk[2:length(GAwk)],TCDmm=TCDmm[2:length(TCDmm)])

      GAwk=rep(NA,1)
      TCDmm=rep(NA,1)
      cumul<-cumsum(xx$n_obs)

      for (i in 1:dim(xx)[1]) {
      GAwk<-c( GAwk,rep(xx$Menstrual_GA_weeks[i],xx$n_obs[i]) )
      TCDmm<-c(TCDmm,
round(rnorm(n=xx$n_obs[i],mean=xx$TCD_mm_mean[i],sd=xx$TCD_mm_sd[i]),1) )}

nig2c<-data.frame(GAwk=GAwk[2:length(GAwk)],TCDmm=TCDmm[2:length(TCDmm)])


xx<-imp3

      GAwk=rep(NA,1)
      TCDmm=rep(NA,1)
      cumul<-cumsum(xx$n_obs)

      for (i in 1:dim(xx)[1]) {
      GAwk<-c( GAwk,rep(xx$Menstrual_GA_weeks[i],xx$n_obs[i]) )
      TCDmm<-c(TCDmm,
round(rnorm(n=xx$n_obs[i],mean=xx$TCD_mm_mean[i],sd=xx$TCD_mm_sd[i]),1) )}

nig3a<-data.frame(GAwk=GAwk[2:length(GAwk)],TCDmm=TCDmm[2:length(TCDmm)])

      GAwk=rep(NA,1)
      TCDmm=rep(NA,1)
      cumul<-cumsum(xx$n_obs)

      for (i in 1:dim(xx)[1]) {
      GAwk<-c( GAwk,rep(xx$Menstrual_GA_weeks[i],xx$n_obs[i]) )
      TCDmm<-c(TCDmm,
round(rnorm(n=xx$n_obs[i],mean=xx$TCD_mm_mean[i],sd=xx$TCD_mm_sd[i]),1) )}

nig3b<-data.frame(GAwk=GAwk[2:length(GAwk)],TCDmm=TCDmm[2:length(TCDmm)])
      GAwk=rep(NA,1)
      TCDmm=rep(NA,1)
      cumul<-cumsum(xx$n_obs)

      for (i in 1:dim(xx)[1]) {
      GAwk<-c( GAwk,rep(xx$Menstrual_GA_weeks[i],xx$n_obs[i]) )
      TCDmm<-c(TCDmm,
round(rnorm(n=xx$n_obs[i],mean=xx$TCD_mm_mean[i],sd=xx$TCD_mm_sd[i]),1) )}

nig3c<-data.frame(GAwk=GAwk[2:length(GAwk)],TCDmm=TCDmm[2:length(TCDmm)])


xx<-imp4

      GAwk=rep(NA,1)
      TCDmm=rep(NA,1)
      cumul<-cumsum(xx$n_obs)

      for (i in 1:dim(xx)[1]) {
      GAwk<-c( GAwk,rep(xx$Menstrual_GA_weeks[i],xx$n_obs[i]) )
      TCDmm<-c(TCDmm,
round(rnorm(n=xx$n_obs[i],mean=xx$TCD_mm_mean[i],sd=xx$TCD_mm_sd[i]),1) )}

inda<-data.frame(GAwk=GAwk[2:length(GAwk)],TCDmm=TCDmm[2:length(TCDmm)])
```

```
      GAwk=rep(NA,1)
      TCDmm=rep(NA,1)
      cumul<-cumsum(xx$n_obs)

      for (i in 1:dim(xx)[1]) {
      GAwk<-c( GAwk,rep(xx$Menstrual_GA_weeks[i],xx$n_obs[i]) )
      TCDmm<-c(TCDmm,
round(rnorm(n=xx$n_obs[i],mean=xx$TCD_mm_mean[i],sd=xx$TCD_mm_sd[i]),1) )}

indb<-data.frame(GAwk=GAwk[2:length(GAwk)],TCDmm=TCDmm[2:length(TCDmm)])

      GAwk=rep(NA,1)
      TCDmm=rep(NA,1)
      cumul<-cumsum(xx$n_obs)

      for (i in 1:dim(xx)[1]) {
      GAwk<-c( GAwk,rep(xx$Menstrual_GA_weeks[i],xx$n_obs[i]) )
      TCDmm<-c(TCDmm,
round(rnorm(n=xx$n_obs[i],mean=xx$TCD_mm_mean[i],sd=xx$TCD_mm_sd[i]),1) )}

indc<-data.frame(GAwk=GAwk[2:length(GAwk)],TCDmm=TCDmm[2:length(TCDmm)])


cknig1<-
data.frame(GAwk=nig1a$GAwk,TCD1=nig1a$TCD,TCD2=nig1b$TCD,TCD3=nig1c$TCD)
cknig2<-
data.frame(GAwk=nig2a$GAwk,TCD1=nig2a$TCD,TCD2=nig2b$TCD,TCD3=nig2c$TCD)
cknig3<-
data.frame(GAwk=nig3a$GAwk,TCD1=nig3a$TCD,TCD2=nig3b$TCD,TCD3=nig3c$TCD)
ckind<-data.frame(GAwk=inda$GAwk,TCD1=inda$TCD,TCD2=indb$TCD,TCD3=indc$TCD)


library("writexl")

write_xlsx(cknig1, "PATH\\nig1 x3.xlsx")
write_xlsx(cknig2, "PATH\\nig2 x3.xlsx")
write_xlsx(cknig3, "PATH\\nig3 x3.xlsx")
write_xlsx(ckind, "PATH\\ind x3.xlsx")


### LOAD:

library("readxl")

ckNIG1 = read_excel("PATH\\check simul process.xlsx", sheet="nig1")
ckNIG2 = read_excel("PATH\\check simul process.xlsx", sheet="nig2")
ckNIG3 = read_excel("PATH\\check simul process.xlsx", sheet="nig3")
ckIND = read_excel("PATH\\check simul process.xlsx", sheet="ind")

ckNIG1<-data.frame(ckNIG1)
ckNIG2<-data.frame(ckNIG2)
ckNIG3<-data.frame(ckNIG3)
ckIND<-data.frame(ckIND)


### DIFFERENT DATA:


xx2<-ckNIG1
plot(xx2[,2]-xx2[,3],pch=19,ylab="Difference between simulated data
points",xlab="N")
points(xx2[,2]-xx2[,4],pch=19,col=2)
points(xx2[,3]-xx2[,4],pch=19,col=3)

xx2<-ckNIG2
```

```
plot(xx2[,2]-xx2[,3],pch=19,ylab="Difference between simulated data
points",xlab="N")
points(xx2[,2]-xx2[,4],pch=19,col=2)
points(xx2[,3]-xx2[,4],pch=19,col=3)

xx2<-ckNIG3
plot(xx2[,2]-xx2[,3],pch=19,ylab="Difference between simulated data
points",xlab="N")
points(xx2[,2]-xx2[,4],pch=19,col=2)
points(xx2[,3]-xx2[,4],pch=19,col=3)

xx2<-ckIND
plot(xx2[,2]-xx2[,3],pch=19,ylab="Difference between simulated data
points",xlab="N")
points(xx2[,2]-xx2[,4],pch=19,col=2)
points(xx2[,3]-xx2[,4],pch=19,col=3)



### DATA CONSISTENT WITH ORIGINAL SUMMARY STATISTICS:

xx3<-ckNIG1
ck1<-data.frame(cbind(
imp1[,1:3],
round(tapply(xx3$TCD1,xx3$GAwk,mean),1),
round(tapply(xx3$TCD1,xx3$GAwk,sd),1),
round(tapply(xx3$TCD2,xx3$GAwk,mean),1),
round(tapply(xx3$TCD2,xx3$GAwk,sd),1),
round(tapply(xx3$TCD3,xx3$GAwk,mean),1),
round(tapply(xx3$TCD3,xx3$GAwk,sd),1)
))

xx3<-ckNIG2
ck2<-data.frame(cbind(
imp2[,1:3],
round(tapply(xx3$TCD1,xx3$GAwk,mean),1),
round(tapply(xx3$TCD1,xx3$GAwk,sd),1),
round(tapply(xx3$TCD2,xx3$GAwk,mean),1),
round(tapply(xx3$TCD2,xx3$GAwk,sd),1),
round(tapply(xx3$TCD3,xx3$GAwk,mean),1),
round(tapply(xx3$TCD3,xx3$GAwk,sd),1)
))

xx3<-ckNIG3
ck3<-data.frame(cbind(
imp3[,1:3],
round(tapply(xx3$TCD1,xx3$GAwk,mean),1),
round(tapply(xx3$TCD1,xx3$GAwk,sd),1),
round(tapply(xx3$TCD2,xx3$GAwk,mean),1),
round(tapply(xx3$TCD2,xx3$GAwk,sd),1),
round(tapply(xx3$TCD3,xx3$GAwk,mean),1),
round(tapply(xx3$TCD3,xx3$GAwk,sd),1)
))

xx3<-ckIND
ck4<-data.frame(cbind(
imp4[,1:3],
round(tapply(xx3$TCD1,xx3$GAwk,mean),1),
round(tapply(xx3$TCD1,xx3$GAwk,sd),1),
round(tapply(xx3$TCD2,xx3$GAwk,mean),1),
round(tapply(xx3$TCD2,xx3$GAwk,sd),1),
round(tapply(xx3$TCD3,xx3$GAwk,mean),1),
round(tapply(xx3$TCD3,xx3$GAwk,sd),1)
))
```

```
library("writexl")

write_xlsx(ck1, "PATH\\ck1.xlsx")
write_xlsx(ck2, "PATH\\ck2.xlsx")
write_xlsx(ck3, "PATH\\ck3.xlsx")
write_xlsx(ck4, "PATH\\ck4.xlsx")
```

```
################ ANALYSIS OF SIMULATED DATA:

rm(list=ls(all=TRUE))

library("readxl")

imp = read_excel("PATH\\simulated_data.xlsx", sheet="Sheet1")

dati<-data.frame(imp)
dati$GA_est_davies<-c ( 0.470*dati$TCDmm + 13.162)
dati$GA_est_nig1<-c ( 0.557*dati$TCDmm + 8.840) #not used
dati$GA_est_nig3<-c ( 0.583*dati$TCDmm + 7.931) #not used
dati$dif<-dati[,4]-dati[,1]
dati$GA_est<-dati$GA_est_davies


#load functions from "NIHMS880429-supplement-Supp_code" (Supplementary
Material: Tipton E, Shuster J. A framework for the meta-analysis of Bland-
Altman studies based on a limits of agreement approach. Stat Med. 2017 Oct
15;36(23):3621-3635. doi: 10.1002/sim.7352. PMID: 28664537)

# The framework used the method-of-moments estimator T2 for the between-study
heterogeneity in bias τ2 as described by the original authors (Tipton et al.)

dati2332<-subset(dati,dati$GAwk>=23 & dati$GAwk<=32)
dati3336<-subset(dati,dati$GAwk>=33 & dati$GAwk<=36)
dati3740<-subset(dati,dati$GAwk>=37 & dati$GAwk<=40)

dati2336<-subset(dati,dati$GAwk>=23 & dati$GAwk<=36)
dati2340<-subset(dati,dati$GAwk>=23 & dati$GAwk<=40)


library(BlandAltmanLeh)

xx1<-dati2332
xx1<-dati3336
xx1<-dati2336


dati22<-data.frame(
Study=c(1:4),
n=c( table(xx1$id)  ) ,
bias=c(
mean(xx1$dif[xx1$id=="nig1"]),mean(xx1$dif[xx1$id=="nig2"]),mean(xx1$dif[xx1$id
=="nig3"]),mean(xx1$dif[xx1$id=="ind"])   ),
stddev=c(
sd(xx1$dif[xx1$id=="nig1"]),sd(xx1$dif[xx1$id=="nig2"]),sd(xx1$dif[xx1$id=="nig
3"]),sd(xx1$dif[xx1$id=="ind"])   )
)

dati22$variance<-dati22$stddev*2
dati22$V_bias<-dati22$variance/10
dati22$logs2<-log(dati22$stddev) + 1/(dati22$n-1)
dati22$V_logs2<-2/(dati22$n-1)

out2 <- loa_maker(dati22$bias,dati22$V_bias,dati22$logs2,dati22$V_logs2)
names(out2) <-
c("studies","bias","sd","tau2","LOA_L","LOA_U","CI_Lm","CI_Um","CI_Lr","CI_Ur")



riss<-data.frame(
id=c("nig1","nig2","nig3","ind","all"),
low=NA,bias=NA,up=NA)
```

```
riss[1,2:4]<-
bland.altman.stats(xx1$GA_est[xx1$id=="nig1"],xx1$GAwk[xx1$id=="nig1"])$lines
riss[2,2:4]<-
bland.altman.stats(xx1$GA_est[xx1$id=="nig2"],xx1$GAwk[xx1$id=="nig2"])$lines
riss[3,2:4]<-
bland.altman.stats(xx1$GA_est[xx1$id=="nig3"],xx1$GAwk[xx1$id=="nig3"])$lines
riss[4,2:4]<-
bland.altman.stats(xx1$GA_est[xx1$id=="ind"],xx1$GAwk[xx1$id=="ind"])$lines
riss[5,2:4]<-out2[c(5,2,6)]

ris2332<-riss
round(ris2332[,2:4]*7,1) #days

ris3336<-riss
round(ris3336[,2:4]*7,1) #days



xx1<-dati3740
xx1<-dati2340

dati22<-data.frame(
Study=c(2:4),
n=c( table(xx1$id  )[2:4]) ,
bias=c(
mean(xx1$dif[xx1$id=="nig2"]),mean(xx1$dif[xx1$id=="nig3"]),mean(xx1$dif[xx1$id
=="ind"])   ),
stddev=c(
sd(xx1$dif[xx1$id=="nig2"]),sd(xx1$dif[xx1$id=="nig3"]),sd(xx1$dif[xx1$id=="ind
"])   )
)

dati22$variance<-dati22$stddev*2
dati22$V_bias<-dati22$variance/10
dati22$logs2<-log(dati22$stddev) + 1/(dati22$n-1)
dati22$V_logs2<-2/(dati22$n-1)

out2 <- loa_maker(dati22$bias,dati22$V_bias,dati22$logs2,dati22$V_logs2)
names(out2) <-
c("studies","bias","sd","tau2","LOA_L","LOA_U","CI_Lm","CI_Um","CI_Lr","CI_Ur")

riss<-data.frame(
id=c("nig2","nig3","ind","all"),
low=NA,bias=NA,up=NA)

riss[2,2:4]<-
bland.altman.stats(xx1$GA_est[xx1$id=="nig2"],xx1$GAwk[xx1$id=="nig2"])$lines
riss[3,2:4]<-
bland.altman.stats(xx1$GA_est[xx1$id=="nig3"],xx1$GAwk[xx1$id=="nig3"])$lines
riss[4,2:4]<-
bland.altman.stats(xx1$GA_est[xx1$id=="ind"],xx1$GAwk[xx1$id=="ind"])$lines
riss[5,2:4]<-out2[c(5,2,6)]

ris3740<-riss
round(ris3740[,2:4]*7,1) #days




### calibration plot 23-32 wk:

xx1<-dati2332
```

```
assex<-c(23:32)
par(mfrow=c(2,2))

xx2<-subset(xx1,xx1$id=="nig1")
estt<-round(tapply(xx2$GA_est_davies,xx2$GAwk,mean),1)
plot(assex,estt,xlab="GA (weeks), obs.", ylab="Mean GA (weeks), 
est.",pch=NA,main="Dataset #1")
lines(assex,estt,col="black",lwd=2)
abline(0,1)
xx2<-subset(xx1,xx1$id=="nig3")
estt<-round(tapply(xx2$GA_est_davies,xx2$GAwk,mean),1)
plot(assex,estt,xlab="GA (weeks), obs.", ylab="Mean GA (weeks), 
est.",pch=NA,main="Dataset #2")
lines(assex,estt,col="black",lwd=2)
abline(0,1)
xx2<-subset(xx1,xx1$id=="nig2")
estt<-round(tapply(xx2$GA_est_davies,xx2$GAwk,mean),1)
plot(assex,estt,xlab="GA (weeks), obs.", ylab="Mean GA (weeks), 
est.",pch=NA,main="Dataset #3")
lines(assex,estt,col="black",lwd=2)
abline(0,1)
xx2<-subset(xx1,xx1$id=="ind")
estt<-round(tapply(xx2$GA_est_davies,xx2$GAwk,mean),1)
plot(assex,estt,xlab="GA (weeks), obs.", ylab="Mean GA (weeks), 
est.",pch=NA,main="Dataset #4")
lines(assex,estt,col="black",lwd=2)
abline(0,1)




### Bland-Altman plot 23-32 wk:

xx1<-dati2332

par(mfrow=c(2,2))

xx2<-subset(xx1,xx1$id=="nig1")
plot(xx2$GAwk,c(xx2$GA_est_davies-xx2$GAwk)*7,col="black",pch=16,ylim=c(-
40,40),xlab="GA (weeks), obs.", ylab="GA (days), difference est. vs. obs.", 
main=paste("Dataset #1","\n\n95% LoA -6.8 to 18.5 days"))
abline(h = ris2332[1,2:4]*7, lty=c(2,3,2), lwd=c(2,2,2))

xx2<-subset(xx1,xx1$id=="nig2")
plot(xx2$GAwk,c(xx2$GA_est_davies-xx2$GAwk)*7,col="black",pch=16,ylim=c(-
40,40),xlab="GA (weeks), obs.", ylab="GA (days), difference est. vs. obs.", 
main=paste("Dataset #2","\n\n95% LoA -7.8 to 25.4 days"))
abline(h = ris2332[2,2:4]*7, lty=c(2,3,2), lwd=c(2,2,2))

xx2<-subset(xx1,xx1$id=="nig3")
plot(xx2$GAwk,c(xx2$GA_est_davies-xx2$GAwk)*7,col="black",pch=16,ylim=c(-
40,40),xlab="GA (weeks), obs.", ylab="GA (days), difference est. vs. obs.", 
main=paste("Dataset #3","\n\n95% LoA -1.1 to 15.4 days"))
abline(h = ris2332[3,2:4]*7, lty=c(2,3,2), lwd=c(2,2,2))

xx2<-subset(xx1,xx1$id=="ind")
plot(xx2$GAwk,c(xx2$GA_est_davies-xx2$GAwk)*7,col="black",pch=16,ylim=c(-
40,40),xlab="GA (weeks), obs.", ylab="GA (days), difference est. vs. obs.", 
main=paste("Dataset #4","\n\n95% LoA -8.5 to 13.9 days"))
abline(h = ris2332[3,2:4]*7, lty=c(2,3,2), lwd=c(2,2,2))



### calib plot 33-36 wk:
```

```
xx1<-dati3336
assex<-c(33:36)
par(mfrow=c(2,2))

xx2<-subset(xx1,xx1$id=="nig1")
estt<-round(tapply(xx2$GA_est_davies,xx2$GAwk,mean),1)
plot(assex,estt,xlab="GA (weeks), obs.", ylab="Mean GA (weeks),
est.",pch=NA,main="Dataset #1")
lines(assex,estt,col="black",lwd=2)
abline(0,1)
xx2<-subset(xx1,xx1$id=="nig3")
estt<-round(tapply(xx2$GA_est_davies,xx2$GAwk,mean),1)
plot(assex,estt,xlab="GA (weeks), obs.", ylab="Mean GA (weeks),
est.",pch=NA,main="Dataset #2")
lines(assex,estt,col="black",lwd=2)
abline(0,1)
xx2<-subset(xx1,xx1$id=="nig2")
estt<-round(tapply(xx2$GA_est_davies,xx2$GAwk,mean),1)
plot(assex,estt,xlab="GA (weeks), obs.", ylab="Mean GA (weeks),
est.",pch=NA,main="Dataset #3")
lines(assex,estt,col="black",lwd=2)
abline(0,1)
xx2<-subset(xx1,xx1$id=="ind")
estt<-round(tapply(xx2$GA_est_davies,xx2$GAwk,mean),1)
plot(assex,estt,xlab="GA (weeks), obs.", ylab="Mean GA (weeks),
est.",pch=NA,main="Dataset #4")
lines(assex,estt,col="black",lwd=2)
abline(0,1)


### Bland-Altman plot 33-36 wk:

xx1<-dati3336

par(mfrow=c(2,2))

xx2<-subset(xx1,xx1$id=="nig1")
plot(xx2$GAwk,c(xx2$GA_est_davies-xx2$GAwk)*7,col="black",pch=16,ylim=c(-
40,40),xlab="GA (weeks), obs.", ylab="GA (days), difference est. vs. obs.",
main=paste("Dataset #1","\n\n95% LoA -17.0 to 18.1 days"))
abline(h = ris3336[1,2:4]*7, lty=c(2,3,2), lwd=c(2,2,2))

xx2<-subset(xx1,xx1$id=="nig2")
plot(xx2$GAwk,c(xx2$GA_est_davies-xx2$GAwk)*7,col="black",pch=16,ylim=c(-
40,40),xlab="GA (weeks), obs.", ylab="GA (days), difference est. vs. obs.",
main=paste("Dataset #2","\n\n95% LoA -16.3 to 18.5 days"))
abline(h = ris3336[2,2:4]*7, lty=c(2,3,2), lwd=c(2,2,2))

xx2<-subset(xx1,xx1$id=="nig3")
plot(xx2$GAwk,c(xx2$GA_est_davies-xx2$GAwk)*7,col="black",pch=16,ylim=c(-
40,40),xlab="GA (weeks), obs.", ylab="GA (days), difference est. vs. obs.",
main=paste("Dataset #3","\n\n95% LoA -9.4 to 10.5 days"))
abline(h = ris3336[3,2:4]*7, lty=c(2,3,2), lwd=c(2,2,2))

xx2<-subset(xx1,xx1$id=="ind")
plot(xx2$GAwk,c(xx2$GA_est_davies-xx2$GAwk)*7,col="black",pch=16,ylim=c(-
40,40),xlab="GA (weeks), obs.", ylab="GA (days), difference est. vs. obs.",
main=paste("Dataset #4","\n\n95% LoA -18.7 to 4.2 days"))
abline(h = ris3336[3,2:4]*7, lty=c(2,3,2), lwd=c(2,2,2))


### calib plot 37-40 wk:

xx1<-dati3740
```

```
assex<-c(37:40)
par(mfrow=c(2,2))

#xx2<-subset(xx1,xx1$id=="nig1")
#estt<-round(tapply(xx2$GA_est_davies,xx2$GAwk,mean),1)
#plot(assex,estt,xlab="GA (weeks), obs.", ylab="Mean GA (weeks),
est.",pch=NA,main="Dataset #1")
#lines(assex,estt,col="black",lwd=2)
#abline(0,1)
xx2<-subset(xx1,xx1$id=="nig3")
estt<-round(tapply(xx2$GA_est_davies,xx2$GAwk,mean),1)
plot(assex,estt,xlab="GA (weeks), obs.", ylab="Mean GA (weeks),
est.",pch=NA,main="Dataset #2")
lines(assex,estt,col="black",lwd=2)
abline(0,1)
xx2<-subset(xx1,xx1$id=="nig2")
estt<-round(tapply(xx2$GA_est_davies,xx2$GAwk,mean),1)
plot(assex,estt,xlab="GA (weeks), obs.", ylab="Mean GA (weeks),
est.",pch=NA,main="Dataset #3")
lines(assex,estt,col="black",lwd=2)
abline(0,1)
xx2<-subset(xx1,xx1$id=="ind")
estt<-round(tapply(xx2$GA_est_davies,xx2$GAwk,mean),1)
plot(assex,estt,xlab="GA (weeks), obs.", ylab="Mean GA (weeks),
est.",pch=NA,main="Dataset #4",ylim=c(35,40),xlim=c(35,40))
lines(assex,estt,col="black",lwd=2)
abline(0,1)


### Bland-Altman plot 37-40 wk:

xx1<-dati3740

par(mfrow=c(2,2))

xx2<-subset(xx1,xx1$id=="nig2")
plot(xx2$GAwk,c(xx2$GA_est_davies-xx2$GAwk)*7,col="black",pch=16,ylim=c(-
40,40),xlab="GA (weeks), obs.", ylab="GA (days), difference est. vs. obs.",
main=paste("Dataset #2","\n\n95% LoA -16.3 to 17.5 days"))
abline(h = ris3740[2,2:4]*7, lty=c(2,3,2), lwd=c(2,2,2))

xx2<-subset(xx1,xx1$id=="nig3")
plot(xx2$GAwk,c(xx2$GA_est_davies-xx2$GAwk)*7,col="black",pch=16,ylim=c(-
40,40),xlab="GA (weeks), obs.", ylab="GA (days), difference est. vs. obs.",
main=paste("Dataset #3","\n\n95% LoA -13.0 to 11.7 days"))
abline(h = ris3740[3,2:4]*7, lty=c(2,3,2), lwd=c(2,2,2))

xx2<-subset(xx1,xx1$id=="ind")
plot(xx2$GAwk,c(xx2$GA_est_davies-xx2$GAwk)*7,col="black",pch=16,ylim=c(-
40,40),xlab="GA (weeks), obs.", ylab="GA (days), difference est. vs. obs.",
main=paste("Dataset #4","\n\n95% LoA -28.2 to -1.2 days"))
abline(h = ris3740[3,2:4]*7, lty=c(2,3,2), lwd=c(2,2,2))
```

```
### CLASSIFICATION ANALYSES:

dati$GAwk_cat<-ifelse(dati$GAwk<28,1,ifelse(dati$GAwk>=28 &
dati$GAwk<=31,2,ifelse(dati$GAwk>=32 & dati$GAwk<=36,3,4)))
dati$GAest_cat<-ifelse(dati$GA_est<28,1,ifelse(dati$GA_est>=28 &
dati$GA_est<=31,2,ifelse(dati$GA_est>=32 & dati$GA_est<=36,3,4)))


#table(dati$GAest_cat==1,dati$GAwk_cat==1,dati$id)

dati_cat1<-
data.frame(id=1:4,matrix(table(dati$GAest_cat==1,dati$GAwk_cat==1,dati$id),ncol
=4,byrow=T))
dati_cat2<-
data.frame(id=1:4,matrix(table(dati$GAest_cat==2,dati$GAwk_cat==2,dati$id),ncol
=4,byrow=T))
dati_cat3<-
data.frame(id=1:4,matrix(table(dati$GAest_cat==3,dati$GAwk_cat==3,dati$id),ncol
=4,byrow=T))
dati_cat4<-
data.frame(id=1:4,matrix(table(dati$GAest_cat==4,dati$GAwk_cat==4,dati$id),ncol
=4,byrow=T))

names(dati_cat1)[2:5]<-c("TN","FP","FN","TP")
names(dati_cat2)[2:5]<-c("TN","FP","FN","TP")
names(dati_cat3)[2:5]<-c("TN","FP","FN","TP")
names(dati_cat4)[2:5]<-c("TN","FP","FN","TP")

require("mada")

#summary(madauni(dati_cat1, type = "posLR"))
round(summary(madauni(dati_cat1, type = "posLR"))$CIcoef[1,],2)
round(summary(madauni(dati_cat1, type = "negLR"))$CIcoef[1,],2)
round(summary(madauni(dati_cat2, type = "posLR"))$CIcoef[1,],2)
round(summary(madauni(dati_cat2, type = "negLR"))$CIcoef[1,],2)
round(summary(madauni(dati_cat3, type = "posLR"))$CIcoef[1,],2)
round(summary(madauni(dati_cat3, type = "negLR"))$CIcoef[1,],2)
round(summary(madauni(dati_cat4, type = "posLR"))$CIcoef[1,],2)
round(summary(madauni(dati_cat4, type = "negLR"))$CIcoef[1,],2)
```

```
### SENSITIVITY ANALYSES:

set.seed(2179)

#niter is not used in runif, rsn, sim_ln_mean_sd functions


#uniform distribution:

xx<-imp1
      GAwk=rep(NA,1)
      TCDmm=rep(NA,1)
      cumul<-cumsum(xx$n_obs)
      for (i in 1:dim(xx)[1]) {
      GAwk<-c( GAwk,rep(xx$Menstrual_GA_weeks[i],xx$n_obs[i]) )
      TCDmm<-c(TCDmm, round(runif(n=xx$n_obs[i],min=xx$TCD_mm_mean[i]-
xx$TCD_mm_sd[i]*sqrt(3),max=xx$TCD_mm_mean[i]+xx$TCD_mm_sd[i]*sqrt(3) ),1) )}
nig1<-data.frame(GAwk=GAwk[2:length(GAwk)],TCDmm=TCDmm[2:length(TCDmm)])

xx<-imp2
      GAwk=rep(NA,1)
      TCDmm=rep(NA,1)
      cumul<-cumsum(xx$n_obs)
      for (i in 1:dim(xx)[1]) {
      GAwk<-c( GAwk,rep(xx$Menstrual_GA_weeks[i],xx$n_obs[i]) )
      TCDmm<-c(TCDmm, round(runif(n=xx$n_obs[i],min=xx$TCD_mm_mean[i]-
xx$TCD_mm_sd[i]*sqrt(3),max=xx$TCD_mm_mean[i]+xx$TCD_mm_sd[i]*sqrt(3) ),1) )}
nig2<-data.frame(GAwk=GAwk[2:length(GAwk)],TCDmm=TCDmm[2:length(TCDmm)])

xx<-imp3
      GAwk=rep(NA,1)
      TCDmm=rep(NA,1)
      cumul<-cumsum(xx$n_obs)
      for (i in 1:dim(xx)[1]) {
      GAwk<-c( GAwk,rep(xx$Menstrual_GA_weeks[i],xx$n_obs[i]) )
      TCDmm<-c(TCDmm, round(runif(n=xx$n_obs[i],min=xx$TCD_mm_mean[i]-
xx$TCD_mm_sd[i]*sqrt(3),max=xx$TCD_mm_mean[i]+xx$TCD_mm_sd[i]*sqrt(3) ),1) )}
nig3<-data.frame(GAwk=GAwk[2:length(GAwk)],TCDmm=TCDmm[2:length(TCDmm)])

xx<-imp4
      GAwk=rep(NA,1)
      TCDmm=rep(NA,1)
      cumul<-cumsum(xx$n_obs)
      for (i in 1:dim(xx)[1]) {
      GAwk<-c( GAwk,rep(xx$Menstrual_GA_weeks[i],xx$n_obs[i]) )
      TCDmm<-c(TCDmm, round(runif(n=xx$n_obs[i],min=xx$TCD_mm_mean[i]-
xx$TCD_mm_sd[i]*sqrt(3),max=xx$TCD_mm_mean[i]+xx$TCD_mm_sd[i]*sqrt(3) ),1) )}
ind<-data.frame(GAwk=GAwk[2:length(GAwk)],TCDmm=TCDmm[2:length(TCDmm)])

nig1$id<-"nig1"
nig2$id<-"nig2"
nig3$id<-"nig3"
ind$id<-"ind"

sim_unif<-data.frame(rbind(nig1,nig2,nig3,ind))

dati<-sim_unif

dati$GA_est<-c(0.470*dati$TCDmm + 13.162)
dati$dif<-dati[,4]-dati[,1]

dati2332<-subset(dati,dati$GAwk>=23 & dati$GAwk<=32)
dati3336<-subset(dati,dati$GAwk>=33 & dati$GAwk<=36)
dati3740<-subset(dati,dati$GAwk>=37 & dati$GAwk<=40)
```

```
dati2336<-subset(dati,dati$GAwk>=23 & dati$GAwk<=36)
dati2340<-subset(dati,dati$GAwk>=23 & dati$GAwk<=40)


library(BlandAltmanLeh)

xx1<-dati2332
xx1<-dati3336
xx1<-dati2336


dati22<-data.frame(
Study=c(1:4),
n=c( table(xx1$id)  ) ,
bias=c(
mean(xx1$dif[xx1$id=="nig1"]),mean(xx1$dif[xx1$id=="nig2"]),mean(xx1$dif[xx1$id
=="nig3"]),mean(xx1$dif[xx1$id=="ind"])   ),
stddev=c(
sd(xx1$dif[xx1$id=="nig1"]),sd(xx1$dif[xx1$id=="nig2"]),sd(xx1$dif[xx1$id=="nig
3"]),sd(xx1$dif[xx1$id=="ind"])   )
)

dati22$variance<-dati22$stddev*2
dati22$V_bias<-dati22$variance/10
dati22$logs2<-log(dati22$stddev) + 1/(dati22$n-1)
dati22$V_logs2<-2/(dati22$n-1)

out2 <- loa_maker(dati22$bias,dati22$V_bias,dati22$logs2,dati22$V_logs2)
names(out2) <-
c("studies","bias","sd","tau2","LOA_L","LOA_U","CI_Lm","CI_Um","CI_Lr","CI_Ur")
round(out2[c(5,2,6)]*7,1) #days


xx1<-dati3740
xx1<-dati2340

dati22<-data.frame(
Study=c(2:4),
n=c( table(xx1$id  )[2:4]) ,
bias=c(
mean(xx1$dif[xx1$id=="nig2"]),mean(xx1$dif[xx1$id=="nig3"]),mean(xx1$dif[xx1$id
=="ind"])   ),
stddev=c(
sd(xx1$dif[xx1$id=="nig2"]),sd(xx1$dif[xx1$id=="nig3"]),sd(xx1$dif[xx1$id=="ind
"])   )
)

dati22$variance<-dati22$stddev*2
dati22$V_bias<-dati22$variance/10
dati22$logs2<-log(dati22$stddev) + 1/(dati22$n-1)
dati22$V_logs2<-2/(dati22$n-1)

out2 <- loa_maker(dati22$bias,dati22$V_bias,dati22$logs2,dati22$V_logs2)
names(out2) <-
c("studies","bias","sd","tau2","LOA_L","LOA_U","CI_Lm","CI_Um","CI_Lr","CI_Ur")
round(out2[c(5,2,6)]*7,1) #days




#skewed normal distribution:

#install.packages("sn")
```

```
library(sn)

xx<-imp1
	GAwk=rep(NA,1)
	TCDmm=rep(NA,1)
	cumul<-cumsum(xx$n_obs)
	for (i in 1:dim(xx)[1]) {
	GAwk<-c( GAwk,rep(xx$Menstrual_GA_weeks[i],xx$n_obs[i]) )
	TCDmm<-c(TCDmm, round(rsn(n=xx$n_obs[i], dp= cp2dp(
c(mean=xx$TCD_mm_mean[i],sd=xx$TCD_mm_sd[i], skewness=0.7), family="SN") ),1)
)}
nig1<-data.frame(GAwk=GAwk[2:length(GAwk)],TCDmm=TCDmm[2:length(TCDmm)])

xx<-imp2
	GAwk=rep(NA,1)
	TCDmm=rep(NA,1)
	cumul<-cumsum(xx$n_obs)
	for (i in 1:dim(xx)[1]) {
	GAwk<-c( GAwk,rep(xx$Menstrual_GA_weeks[i],xx$n_obs[i]) )
	TCDmm<-c(TCDmm, round(rsn(n=xx$n_obs[i], dp= cp2dp(
c(mean=xx$TCD_mm_mean[i],sd=xx$TCD_mm_sd[i], skewness=0.7), family="SN") ),1)
)}
nig2<-data.frame(GAwk=GAwk[2:length(GAwk)],TCDmm=TCDmm[2:length(TCDmm)])

xx<-imp3
	GAwk=rep(NA,1)
	TCDmm=rep(NA,1)
	cumul<-cumsum(xx$n_obs)
	for (i in 1:dim(xx)[1]) {
	GAwk<-c( GAwk,rep(xx$Menstrual_GA_weeks[i],xx$n_obs[i]) )
	TCDmm<-c(TCDmm, round(rsn(n=xx$n_obs[i], dp= cp2dp(
c(mean=xx$TCD_mm_mean[i],sd=xx$TCD_mm_sd[i], skewness=0.7), family="SN") ),1)
)}
nig3<-data.frame(GAwk=GAwk[2:length(GAwk)],TCDmm=TCDmm[2:length(TCDmm)])

xx<-imp4
	GAwk=rep(NA,1)
	TCDmm=rep(NA,1)
	cumul<-cumsum(xx$n_obs)
	for (i in 1:dim(xx)[1]) {
	GAwk<-c( GAwk,rep(xx$Menstrual_GA_weeks[i],xx$n_obs[i]) )
	TCDmm<-c(TCDmm, round(rsn(n=xx$n_obs[i], dp= cp2dp(
c(mean=xx$TCD_mm_mean[i],sd=xx$TCD_mm_sd[i], skewness=0.7), family="SN") ),1)
)}
ind<-data.frame(GAwk=GAwk[2:length(GAwk)],TCDmm=TCDmm[2:length(TCDmm)])

nig1$id<-"nig1"
nig2$id<-"nig2"
nig3$id<-"nig3"
ind$id<-"ind"

sim_skewed<-data.frame(rbind(nig1,nig2,nig3,ind))



dati<-sim_skewed

dati$GA_est<-c(0.470*dati$TCDmm + 13.162)
dati$dif<-dati[,4]-dati[,1]

dati2332<-subset(dati,dati$GAwk>=23 & dati$GAwk<=32)
dati3336<-subset(dati,dati$GAwk>=33 & dati$GAwk<=36)
dati3740<-subset(dati,dati$GAwk>=37 & dati$GAwk<=40)
```

```
dati2336<-subset(dati,dati$GAwk>=23 & dati$GAwk<=36)
dati2340<-subset(dati,dati$GAwk>=23 & dati$GAwk<=40)



library(BlandAltmanLeh)

xx1<-dati2332
xx1<-dati3336
xx1<-dati2336


dati22<-data.frame(
Study=c(1:4),
n=c( table(xx1$id)  ) ,
bias=c(
mean(xx1$dif[xx1$id=="nig1"]),mean(xx1$dif[xx1$id=="nig2"]),mean(xx1$dif[xx1$id
=="nig3"]),mean(xx1$dif[xx1$id=="ind"])   ),
stddev=c(
sd(xx1$dif[xx1$id=="nig1"]),sd(xx1$dif[xx1$id=="nig2"]),sd(xx1$dif[xx1$id=="nig
3"]),sd(xx1$dif[xx1$id=="ind"])   )
)

dati22$variance<-dati22$stddev*2
dati22$V_bias<-dati22$variance/10
dati22$logs2<-log(dati22$stddev) + 1/(dati22$n-1)
dati22$V_logs2<-2/(dati22$n-1)

out2 <- loa_maker(dati22$bias,dati22$V_bias,dati22$logs2,dati22$V_logs2)
names(out2) <-
c("studies","bias","sd","tau2","LOA_L","LOA_U","CI_Lm","CI_Um","CI_Lr","CI_Ur")
round(out2[c(5,2,6)]*7,1) #days


xx1<-dati3740
xx1<-dati2340

dati22<-data.frame(
Study=c(2:4),
n=c( table(xx1$id  )[2:4]) ,
bias=c(
mean(xx1$dif[xx1$id=="nig2"]),mean(xx1$dif[xx1$id=="nig3"]),mean(xx1$dif[xx1$id
=="ind"])   ),
stddev=c(
sd(xx1$dif[xx1$id=="nig2"]),sd(xx1$dif[xx1$id=="nig3"]),sd(xx1$dif[xx1$id=="ind
"])   )
)

dati22$variance<-dati22$stddev*2
dati22$V_bias<-dati22$variance/10
dati22$logs2<-log(dati22$stddev) + 1/(dati22$n-1)
dati22$V_logs2<-2/(dati22$n-1)

out2 <- loa_maker(dati22$bias,dati22$V_bias,dati22$logs2,dati22$V_logs2)
names(out2) <-
c("studies","bias","sd","tau2","LOA_L","LOA_U","CI_Lm","CI_Um","CI_Lr","CI_Ur")
round(out2[c(5,2,6)]*7,1) #days



#heavy tails:


sim_ln_mean_sd <- function(n, mean, sd) {
      if (mean <= 0) stop("Mean>0 for log-normal distribution")
```

```
      log_sd<-sqrt(log(1+( (sd^2)/(mean^2))))
      log_mean<-log(mean)-(log_sd^2 /2)
    sim<- rlnorm(n, log_mean, log_sd)
  return(sim)
}




xx<-imp1
      GAwk=rep(NA,1)
      TCDmm=rep(NA,1)
      cumul<-cumsum(xx$n_obs)
      for (i in 1:dim(xx)[1]) {
      GAwk<-c( GAwk,rep(xx$Menstrual_GA_weeks[i],xx$n_obs[i]) )
      TCDmm<-c(TCDmm, round( sim_ln_mean_sd(n=xx$n_obs[i],
mean=xx$TCD_mm_mean[i], sd=xx$TCD_mm_sd[i]),1) )}
nig1<-data.frame(GAwk=GAwk[2:length(GAwk)],TCDmm=TCDmm[2:length(TCDmm)])

xx<-imp2
      GAwk=rep(NA,1)
      TCDmm=rep(NA,1)
      cumul<-cumsum(xx$n_obs)
      for (i in 1:dim(xx)[1]) {
      GAwk<-c( GAwk,rep(xx$Menstrual_GA_weeks[i],xx$n_obs[i]) )
      TCDmm<-c(TCDmm, round( sim_ln_mean_sd(n=xx$n_obs[i],
mean=xx$TCD_mm_mean[i], sd=xx$TCD_mm_sd[i]),1) )}
nig2<-data.frame(GAwk=GAwk[2:length(GAwk)],TCDmm=TCDmm[2:length(TCDmm)])

xx<-imp3
      GAwk=rep(NA,1)
      TCDmm=rep(NA,1)
      cumul<-cumsum(xx$n_obs)
      for (i in 1:dim(xx)[1]) {
      GAwk<-c( GAwk,rep(xx$Menstrual_GA_weeks[i],xx$n_obs[i]) )
      TCDmm<-c(TCDmm, round( sim_ln_mean_sd(n=xx$n_obs[i],
mean=xx$TCD_mm_mean[i], sd=xx$TCD_mm_sd[i]),1) )}
nig3<-data.frame(GAwk=GAwk[2:length(GAwk)],TCDmm=TCDmm[2:length(TCDmm)])

xx<-imp4
      GAwk=rep(NA,1)
      TCDmm=rep(NA,1)
      cumul<-cumsum(xx$n_obs)
      for (i in 1:dim(xx)[1]) {
      GAwk<-c( GAwk,rep(xx$Menstrual_GA_weeks[i],xx$n_obs[i]) )
      TCDmm<-c(TCDmm, round( sim_ln_mean_sd(n=xx$n_obs[i],
mean=xx$TCD_mm_mean[i], sd=xx$TCD_mm_sd[i]),1) )}
ind<-data.frame(GAwk=GAwk[2:length(GAwk)],TCDmm=TCDmm[2:length(TCDmm)])

nig1$id<-"nig1"
nig2$id<-"nig2"
nig3$id<-"nig3"
ind$id<-"ind"

sim_heavy<-data.frame(rbind(nig1,nig2,nig3,ind))



dati<-sim_heavy

dati$GA_est<-c(0.470*dati$TCDmm + 13.162)
dati$dif<-dati[,4]-dati[,1]

dati2332<-subset(dati,dati$GAwk>=23 & dati$GAwk<=32)
```

```
dati3336<-subset(dati,dati$GAwk>=33 & dati$GAwk<=36)
dati3740<-subset(dati,dati$GAwk>=37 & dati$GAwk<=40)

dati2336<-subset(dati,dati$GAwk>=23 & dati$GAwk<=36)
dati2340<-subset(dati,dati$GAwk>=23 & dati$GAwk<=40)



library(BlandAltmanLeh)

xx1<-dati2332
xx1<-dati3336
xx1<-dati2336


dati22<-data.frame(
Study=c(1:4),
n=c( table(xx1$id)  ) ,
bias=c(
mean(xx1$dif[xx1$id=="nig1"]),mean(xx1$dif[xx1$id=="nig2"]),mean(xx1$dif[xx1$id
=="nig3"]),mean(xx1$dif[xx1$id=="ind"])   ),
stddev=c(
sd(xx1$dif[xx1$id=="nig1"]),sd(xx1$dif[xx1$id=="nig2"]),sd(xx1$dif[xx1$id=="nig
3"]),sd(xx1$dif[xx1$id=="ind"])   )
)

dati22$variance<-dati22$stddev*2
dati22$V_bias<-dati22$variance/10
dati22$logs2<-log(dati22$stddev) + 1/(dati22$n-1)
dati22$V_logs2<-2/(dati22$n-1)

out2 <- loa_maker(dati22$bias,dati22$V_bias,dati22$logs2,dati22$V_logs2)
names(out2) <-
c("studies","bias","sd","tau2","LOA_L","LOA_U","CI_Lm","CI_Um","CI_Lr","CI_Ur")
round(out2[c(5,2,6)]*7,1) #days


xx1<-dati3740
xx1<-dati2340

dati22<-data.frame(
Study=c(2:4),
n=c( table(xx1$id  )[2:4]) ,
bias=c(
mean(xx1$dif[xx1$id=="nig2"]),mean(xx1$dif[xx1$id=="nig3"]),mean(xx1$dif[xx1$id
=="ind"])   ),
stddev=c(
sd(xx1$dif[xx1$id=="nig2"]),sd(xx1$dif[xx1$id=="nig3"]),sd(xx1$dif[xx1$id=="ind
"])   )
)

dati22$variance<-dati22$stddev*2
dati22$V_bias<-dati22$variance/10
dati22$logs2<-log(dati22$stddev) + 1/(dati22$n-1)
dati22$V_logs2<-2/(dati22$n-1)

out2 <- loa_maker(dati22$bias,dati22$V_bias,dati22$logs2,dati22$V_logs2)
names(out2) <-
c("studies","bias","sd","tau2","LOA_L","LOA_U","CI_Lm","CI_Um","CI_Lr","CI_Ur")
round(out2[c(5,2,6)]*7,1) #days
```
