# Supplementary material for: Postnatal gestational age determination by ultrasonographic measurement of transverse cerebellar diameter: A simulation study
Source: PLoS One. 2026 Aug 24;21(8):e0356370. doi: 10.1371/journal.pone.0356370 (PMC13502679; doi:10.1371/journal.pone.0356370)
Supplement: Supplementary file 2 [file pone.0356370.s021.pdf]

Padova, July 28, 2026

Manuscript PONE-D-26-12831R1

Dear Sikolia Wanyonyi
Academic Editor
PLOS One

Enclosed please find the revised version of our manuscript entitled "Postnatal Gestational Age Determination by Ultrasonographic Measurement of Transverse Cerebellar Diameter: a Simulation Study".

We thank the Editor and the Reviewers for their comments, and we hope to have satisfactorily answered to all queries.

Best regards,

Andrea Pietravalle,

Doctors with Africa CUAMM, Padua, Italy

Comments to the Author

Reviewer #2:

Some of the suggested corrections were implemented, but the manuscript could be further improved by addressing the following points:

1. Include a brief PRISMA flow diagram showing why only four studies were included, and explain how the selection does not (or does) limit geographic/phenotypic representativeness and generalizability.

Re: We thank the Reviewer for the suggestion and we added the PRISMA flow diagram as Supplementary Figure 1: "Four studies met the inclusion criteria and were used for the simulation process (Supplementary Figure 1). [26-29]" (Results, page 4). In the revised manuscript, we added some considerations about the inclusion criteria limiting the generalizability of the findings to LMICs, and the literature output limiting geographic and phenotypic representativeness: "Overall, the generalizability of the findings may be limited to LMICs due to the inclusion criteria, while geographic and phenotypic representativeness was limited by the literature output that yielded only three studies from Nigeria and one study from India. Population-specific differences in fetal anthropometry and growth patterns could theoretically affect the performance of a gestational-age prediction model when applied across different settings. Indeed, ethnic differences in TCD growth curves have been reported, suggesting that population-specific reference charts may improve accuracy in some settings. [41,42] However, the original postnatal TCD equation was developed in Australia and subsequently evaluated in an independent cohort from Spain, showing comparable performance despite the different geographic and population settings. [22,23] Nevertheless, some degree of population-specific variability cannot be excluded, thus caution is suggested in the interpretation of our findings that may be integrated by future research using individual-level data from geographically different populations." (Discussion, page 10).

2. Provide the TCD measurement technique used in each source study; discuss how differences in technique may bias TCD and therefore estimated GA. Confirm and state whether measurements were prenatal fetal or postnatal, and discuss the impact.

Re: We thank the Reviewer for the suggestion. We added the TCD measurement technique used in each source study as Supplementary Table 2, with reference in the text of the manuscript: "All four studies employed a basic standard methodological approach to evaluate the TCD during routine prenatal fetal ultrasound (Supplementary Table 2)." (Results, page 4). We added some considerations about differences in technique: "Moreover, we cannot exclude some heterogeneity in TCD measurement technique across the studies, despite the employment of a basic standard methodological approach to evaluate the TCD during routine prenatal fetal ultrasound. [26-29] Such heterogeneity may be due to minor differences in image acquisition, caliper placement, transducer orientation and operator expertise. Since Davies's equation estimates GA using TCD measures [22], these methodological differences may proportionally translate into slightly different GA estimates and contribute to between-study heterogeneity, which is accounted for by the random-effects pooling of the bias." (Discussion, pages 10-11). We also added some considerations about prenatal and postnatal measurements: "It should be underlined that Davies's equation was originally developed using postnatal ultrasound, while all source measurements were prenatal

fetal measurements. [22,26-29] The equation aimed to refine the use of TCD as reliable predictor of GA, and recent evidence demonstrated strong agreement between prenatal and postnatal TCD measurements [24], but prospective validation using real postnatal measurements remains necessary." (Discussion, page 9).

3. Methods: a) Specify niter (number of simulation iterations per study) used in the main analyses.; b) Provide the random seeds and describe exactly how the "skewed" and "heavy-tail" distributions were generated (parameters), or refer to the supplementary material where this is documented; c) Include in the supplementary material: i) executable R code that reproduces each figure and table, (ii) the method used to estimate τ2 (e.g., DerSimonian–Laird, REML), and (iii) heterogeneity metrics (I2 / τ2) and their impact on pooled limits of agreement.

Re: We reported this information (niter, seed, distributions) in the "Supplementary Material - R code" file and added a reference to the supplementary material in the revised manuscript. The code describes how the skewed distribution and heavy-tail distribution were generated and their parameters. In the "Supplementary Material - R code", we specified that "The framework used the method-of-moments estimator T2 for the between-study heterogeneity in bias τ2 as described by the original authors (Tipton et al.).". In the revised manuscript, we reported the heterogeneity metrics τ2 that are produced by the functions of the framework by Tipton et al. (Results, pages 5-6) and we commented about their impact on the findings: "Our simulation-based evaluation suggested that such equation may provide some benefits in estimating GA between 23-32 weeks, which was the original domain of the equation. [22] When the equation was extended beyond the original domain, the between-study heterogeneity increased and the agreement slightly impaired as could be expected. These findings are correlated as higher between-study heterogeneity produces larger agreement intervals [32], and suggests caution in using the equation beyond the original domain." (Discussion, page 8).

4. Expand the Discussion to explicitly acknowledge limitations arising from using algorithm-generated values and the need for clinical validation; state that the true utility of this tool as clinical support will only be known after validation in real clinical settings, and discuss constraints on implementation in different clinical environments (e.g., limited access to ultrasound equipment).

Re: We thank the Reviewer for the suggestion. In the revised manuscript, we added some considerations about the use of algorithm-generated values: "The main limitation of our study is the use of algorithm-generated values to assess the performance of Davies's equation in estimating GA using fetal TCD measurements. [22] Simulated data may oversimplify real-world data, hence failing to replicate patient heterogeneity. In this perspective, our findings provide promising information on the potential application of the equation, but clinical validation remains a paramount to assess its utility in the real world. In addition, the true distribution of TCD may be non-Normal, but the sensitivity analysis suggested comparable GA estimates when different weekly distributions (Normal, uniform, skewed, heavy tail) were used to simulate TCD data. Finally, the quality of the simulation depends on the quality of both assumptions and input data, hence suggesting caution in the interpretation of the results. Unfortunately, original raw data were not provided in the papers, hence we could not use raw data to inform the simulation process or quantify the bias that arose from our simulation process." (Discussion, page 10). We also added some considerations at the end of the Discussion section: "Due to the key limitation of using

simulated data (although derived from actual fetal measurements), the true utility of this tool as clinical support will only be known after validation in real clinical settings and future studies may validate the equation by Davies et al. [22] using TCD measurements from real newborns in low-resource settings where precise GA dating is available. The inherent condition for real-world implementation is clearly the availability of an ultrasound. Despite recent developments of such technology have led to affordable devices and progressive spread in the diagnostics process, local constraints such as limited access to ultrasound equipment and limited expertise can impair the implementation of Davies's equation in the clinical environments of some LMICs." (Discussion, page 11).
