# Supplementary material for: Postnatal gestational age determination by ultrasonographic measurement of transverse cerebellar diameter: A simulation study
Source: PLoS One. 2026 Aug 24;21(8):e0356370. doi: 10.1371/journal.pone.0356370 (PMC13502679; doi:10.1371/journal.pone.0356370)
Supplement: Supplementary file 3 [file pone.0356370.s001.docx]

Table S1. Relevant data extracted from the selected studies. GA: gestational age. NA: not available. SD: standard deviation. TCD: transverse cerebellar diameter.

| Menstru al GA,  weeks | Study #1 | | Study #2 | | Study #3 | | Study #4 | |
| --- | --- | --- | --- | --- | --- | --- | --- | --- |
|  | N of observati  ons | TCD:  mean  (SD) | N of observati  ons | TCD:  mean  (SD) | N of observati  ons | TCD:  mean  (SD) | N of observati  ons | TCD:  mean  (SD) |
| 13 | NA | NA | 11 | 12.8 (0.8) | NA | NA | NA | NA |
| 14 | 15 | 13.3 (0.3) | 14 | 13.6 (1) | NA | NA | NA | NA |
| 15 | 16 | 14.1 (0.7) | 11 | 14.9 (0.8) | NA | NA | NA | NA |
| 16 | 15 | 16.4 (1.3) | 18 | 15.6 (0.7) | 8 | 15.7 (0.2) | NA | NA |
| 17 | 17 | 16.9 (0.8) | 11 | 16.5 (1) | 8 | 17.2 (0.1) | NA | NA |
| 18 | 15 | 18.1 (1.3) | 15 | 18.2 (0.8) | 18 | 18.7 (0.5) | 25 | 18.5 (0.4) |
| 19 | 16 | 19.5 (2.2) | 7 | 19.5 (0.8) | 11 | 21.6 (0.1) | 35 | 20.7 (0.3) |
| 20 | 17 | 21 (3.4) | 15 | 20.7 (0.6) | 8 | 21 (0.5) | 40 | 21.1 (0.2) |
| 21 | 20 | 21.4 (1.7) | 20 | 21.6 (0.9) | 6 | 21.9 (0.2) | 25 | 21.8 (0.2) |
| 22 | 17 | 22.5 (1.5) | 21 | 23.2 (2) | 24 | 22.4 (0.2) | 32 | 22.6 (0.3) |
| 23 | 18 | 24.5 (3.3) | 16 | 24.3 (1.9) | 22 | 24.4 (0.2) | 15 | 24.3 (0.3) |
| 24 | 16 | 25.7 (1.5) | 19 | 26.8 (1.6) | 11 | 24.5 (0.3) | 10 | 25.2 (0.4) |
| 25 | 15 | 27 (1.9) | 18 | 28.5 (1.9) | 12 | 28.9 (0.2) | 8 | 27 (0.3) |
| 26 | 16 | 29.5 (2.1) | 16 | 30.5 (2.8) | 4 | 30.2 (0.5) | 10 | 29.5 (0.4) |
| 27 | 15 | 30.9 (2.4) | 12 | 31.3 (1.5) | 18 | 31.3 (0.5) | 11 | 30.3 (0.4) |
| 28 | 16 | 33.2 (1.3) | 11 | 33.9 (3.1) | 24 | 33.9 (0.6) | 48 | 33.4 (0.5) |
| 29 | 15 | 35.9 (2.8) | 15 | 35.5 (2.7) | 10 | 35.6 (1.5) | 24 | 34.8 (1.2) |
| 30 | 16 | 37.1 (0.5) | 13 | 38.1 (2.6) | 11 | 37.5 (1.4) | 22 | 35.6 (1.5) |
| 31 | 15 | 39.4 (1.1) | 12 | 39.4 (1.8) | 8 | 39.1 (0.5) | 14 | 37.2 (1.5) |
| 32 | 16 | 41.5 (0.6) | 16 | 39.9 (2.3) | 10 | 40.2 (1.1) | 41 | 38.4 (1.6) |
| 33 | 17 | 41.8 (3.9) | 9 | 42.9 (1.9) | 10 | 42.9 (1.4) | 22 | 40.2 (1.5) |
| 34 | 16 | 45.4 (5.4) | 17 | 44.4 (3.2) | 3 | 44.8 (0.5) | 10 | 42.4 (1.5) |
| 35 | 16 | 46.1 (2.6) | 25 | 46.9 (2.2) | 10 | 46.7 (1.5) | 14 | 43.8 (1.6) |
| 36 | 15 | 49.7 (1.2) | 29 | 49.8 (3.4) | 6 | 47.8 (3.8) | 35 | 45.6 (1.8) |
| 37 | 15 | 51.3 (1) | 31 | 50.9 (3.3) | 4 | 50.3 (1.3) | 11 | 47.4 (1.8) |
| 38 | 15 | 52.3 (3.3) | 25 | 53.3 (2.2) | 6 | 51.3 (1.3) | 30 | 48.5 (1.9) |
| 39 | NA | NA | 6 | 54.8 (2.6) | 3 | 57.2 (0.5) | 10 | 50.2 (2.2) |
| 40 | NA | NA | 4 | 58 (0) | 2 | 58.1 (2.3) | 8 | 51.5 (2.5) |
| 41 | NA | NA | 10 | 58.3 (0.5) | NA | NA | NA | NA |
| 42 | NA | NA | 3 | 58.9 (0.2) | NA | NA | NA | NA |
