# Supplementary material for: Postnatal gestational age determination by ultrasonographic measurement of transverse cerebellar diameter: A simulation study
Source: PLoS One. 2026 Aug 24;21(8):e0356370. doi: 10.1371/journal.pone.0356370 (PMC13502679; doi:10.1371/journal.pone.0356370)
Supplement: Supplementary file 4 [file pone.0356370.s002.docx]

Table S2. Description of the technique used in each source study to measure the transverse cerebellar diameter (TCD).

| Study #1 (Adelabu et al.) | The authors calculated the TCD electronically using the scanner. After obtaining the thalamic plane used for biparietal diameter, the transducer was rotated about 30° below the thalamic plane to image the characteristic shape of the cerebellum. The TCD was measured in an “outer‑to‑outer” fashion. |
| --- | --- |
| Study #2 (Adeyekun et al.) | The authors located the fetal cerebellum by first using the horizontal plane of the fetal head to identify the cavum septum pellucidum, and the thalami as landmarks similar to the plane for the biparietal diameter. The transducer was then slightly rotated caudally, to bring the characteristic “butterfly” appearance of the cerebellum into view- The TCD was measured in an “outer‑to‑outer” fashion. |
| Study #3 (Eze et al.) | From the biparietal diameter plane, the authors slightly rotated the probe and moved inferiorly toward the fetal neck to display the anterior horns of lateral ventricles and cavum septum pellucidum anteriorly, while the cerebellum and cisterna magna were visualized posteriorly in the trans-cerebellar plane. The TCD was measured by placing the on-screen caliper of the ultrasound machine at the outer margins of the cerebellum. |
| Study #4 (Kummari et al.) | The authors stated that they measured TCD in addition to routine ultrasonography parameters such as biparietal diameter or head circumference. They displayed the technique in a figure, where TCD was measured in an “outer‑to‑outer” fashion. |
