# Supplementary material for: Postnatal gestational age determination by ultrasonographic measurement of transverse cerebellar diameter: A simulation study
Source: PLoS One. 2026 Aug 24;21(8):e0356370. doi: 10.1371/journal.pone.0356370 (PMC13502679; doi:10.1371/journal.pone.0356370)
Supplement: Supplementary file 5 [file pone.0356370.s003.docx]

Table S3. The simulation process was checked using three repetitions for Study #1, which correctly produced consistent simulated data. GA: gestational age. SD: standard deviation. TCD: transverse cerebellar diameter.

| Menstrual GA weeks | Reported TCD, mm: mean (SD) | Repetition #1: estimated TCD,  mm: mean (SD) | Repetition #2: estimated TCD,  mm: mean (SD) | Repetition #3: estimated TCD,  mm: mean (SD) |
| --- | --- | --- | --- | --- |
| 14 | 13.3 (0.3) | 13.3 (0.2) | 13.3 (0.2) | 13.4 (0.4) |
| 15 | 14.1 (0.7) | 14.2 (0.6) | 13.8 (0.4) | 14.1 (0.5) |
| 16 | 16.4 (1.3) | 16.4 (1) | 15.8 (1.6) | 15.9 (1.4) |
| 17 | 16.9 (0.8) | 16.5 (0.9) | 17 (0.9) | 17.1 (0.7) |
| 18 | 18.1 (1.3) | 17.9 (1.4) | 18.9 (1.4) | 18.2 (1.1) |
| 19 | 19.5 (2.2) | 20.1 (1.8) | 19.5 (1.7) | 18.9 (2.2) |
| 20 | 21 (3.4) | 21.3 (3.3) | 21.3 (2.7) | 20.2 (3.4) |
| 21 | 21.4 (1.7) | 21 (1.7) | 21.1 (1.3) | 21.8 (2) |
| 22 | 22.5 (1.5) | 22.7 (1.5) | 22.3 (1.2) | 22.7 (1.7) |
| 23 | 24.5 (3.3) | 24.6 (3) | 25.3 (3.6) | 24.2 (3.4) |
| 24 | 25.7 (1.5) | 25.1 (1.4) | 26.3 (1.3) | 26.6 (1.6) |
| 25 | 27 (1.9) | 27.2 (1.8) | 27 (1.8) | 26.5 (2.2) |
| 26 | 29.5 (2.1) | 29.9 (2.3) | 30 (1.6) | 29.5 (2.2) |
| 27 | 30.9 (2.4) | 31.9 (2.4) | 31.4 (3) | 30.6 (2.3) |
| 28 | 33.2 (1.3) | 33.1 (1.1) | 32.9 (1.2) | 33.2 (1.3) |
| 29 | 35.9 (2.8) | 35.4 (2.4) | 36.5 (2.4) | 34.9 (3.6) |
| 30 | 37.1 (0.5) | 37.1 (0.5) | 37.1 (0.7) | 37 (0.5) |
| 31 | 39.4 (1.1) | 39.6 (1.1) | 39.1 (1.4) | 39 (1.2) |
| 32 | 41.5 (0.6) | 41.3 (0.7) | 41.7 (0.6) | 41.8 (0.5) |
| 33 | 41.8 (3.9) | 41.2 (4.1) | 42.7 (3.7) | 40.7 (4.2) |
| 34 | 45.4 (5.4) | 45.9 (5.8) | 43.1 (7) | 43.6 (6) |
| 35 | 46.1 (2.6) | 46.4 (2.7) | 46.3 (1.9) | 45.7 (2.4) |
| 36 | 49.7 (1.2) | 49.1 (0.8) | 49.8 (1.3) | 49.6 (1.1) |
| 37 | 51.3 (1) | 50.8 (0.9) | 51.1 (1) | 51.2 (0.8) |
| 38 | 52.3 (3.3) | 53.1 (3.1) | 52.1 (3.5) | 53.2 (3.4) |
