# Supplementary material for: Postnatal gestational age determination by ultrasonographic measurement of transverse cerebellar diameter: A simulation study
Source: PLoS One. 2026 Aug 24;21(8):e0356370. doi: 10.1371/journal.pone.0356370 (PMC13502679; doi:10.1371/journal.pone.0356370)
Supplement: Supplementary file 6 [file pone.0356370.s004.docx]

Table S4. The simulation process was checked using three repetitions for Study #2, which correctly produced consistent simulated data. GA: gestational age. SD: standard deviation. TCD: transverse cerebellar diameter.

| Menstrual GA weeks | Reported TCD, mm: mean (SD) | Repetition #1:  estimated TCD, mm: mean (SD) | Repetition #2:  estimated TCD, mm: mean (SD) | Repetition #3:  estimated TCD, mm: mean (SD) |
| --- | --- | --- | --- | --- |
| 13 | 12.8 (0.8) | 12.5 (0.8) | 13.1 (0.9) | 12.9 (0.9) |
| 14 | 13.6 (1) | 13.5 (1.4) | 13.4 (1.1) | 13.4 (0.8) |
| 15 | 14.9 (0.8) | 15.4 (0.9) | 15 (0.9) | 14.8 (0.5) |
| 16 | 15.6 (0.7) | 15.5 (0.9) | 15.7 (0.7) | 16.1 (0.5) |
| 17 | 16.5 (1) | 16.5 (1.1) | 16.5 (1.1) | 16.5 (0.7) |
| 18 | 18.2 (0.8) | 18.4 (0.7) | 18.5 (1) | 18.4 (1) |
| 19 | 19.5 (0.8) | 19.6 (0.6) | 19.3 (0.6) | 19.6 (0.8) |
| 20 | 20.7 (0.6) | 20.5 (0.3) | 20.8 (0.5) | 20.7 (0.5) |
| 21 | 21.6 (0.9) | 21.4 (1) | 21.7 (0.9) | 21.8 (1.1) |
| 22 | 23.2 (2) | 23.2 (1.4) | 23.7 (2.2) | 23.9 (2.1) |
| 23 | 24.3 (1.9) | 24.5 (1.3) | 24.3 (2.4) | 24.3 (2) |
| 24 | 26.8 (1.6) | 26.6 (1.4) | 26.4 (1.1) | 26.9 (1.2) |
| 25 | 28.5 (1.9) | 27.9 (1.7) | 29.5 (1.8) | 28.6 (1.5) |
| 26 | 30.5 (2.8) | 30.1 (3.1) | 32.5 (2.5) | 29.8 (2.9) |
| 27 | 31.3 (1.5) | 31.1 (1.4) | 32.1 (1.4) | 31.1 (1.7) |
| 28 | 33.9 (3.1) | 32.3 (3.2) | 32.5 (3.4) | 34.5 (2.2) |
| 29 | 35.5 (2.7) | 36.7 (2.5) | 34.9 (2.8) | 35.3 (3) |
| 30 | 38.1 (2.6) | 37.9 (2.4) | 37.3 (3.4) | 38.6 (2.6) |
| 31 | 39.4 (1.8) | 39.6 (1.3) | 39.4 (1.6) | 39.8 (1.6) |
| 32 | 39.9 (2.3) | 39.9 (2.2) | 40.2 (2.7) | 39.8 (3.3) |
| 33 | 42.9 (1.9) | 43.5 (2.2) | 43.4 (2.3) | 43.5 (1.8) |
| 34 | 44.4 (3.2) | 44.8 (3.9) | 44.2 (3.3) | 44.6 (2.4) |
| 35 | 46.9 (2.2) | 47.3 (2.3) | 46.3 (1.9) | 46.9 (1.6) |
| 36 | 49.8 (3.4) | 49 (3.7) | 48.6 (3.7) | 49.2 (3.2) |
| 37 | 50.9 (3.3) | 50.7 (4) | 50.5 (4) | 50.4 (2.3) |
| 38 | 53.3 (2.2) | 52.7 (2.1) | 53 (2.9) | 53.5 (2.4) |
| 39 | 54.8 (2.6) | 55 (2.9) | 54.9 (0.9) | 54 (1.7) |
| 40 | 58 (0) | 58 (0) | 58 (0) | 58 (0) |
| 41 | 58.3 (0.5) | 58.2 (0.4) | 58.6 (0.4) | 58.4 (0.7) |
| 42 | 58.9 (0.2) | 58.8 (0.2) | 58.8 (0.2) | 58.8 (0.2) |
