# Supplementary material for: Postnatal gestational age determination by ultrasonographic measurement of transverse cerebellar diameter: A simulation study
Source: PLoS One. 2026 Aug 24;21(8):e0356370. doi: 10.1371/journal.pone.0356370 (PMC13502679; doi:10.1371/journal.pone.0356370)
Supplement: Supplementary file 7 [file pone.0356370.s005.docx]

Table S5. The simulation process was checked using three repetitions for Study #3, which correctly produced consistent simulated data. GA: gestational age. SD: standard deviation. TCD: transverse cerebellar diameter.

| Menstrual GA weeks | Reported TCD, mm: mean (SD) | Repetition #1: estimated TCD, mm:  mean (SD) | Repetition #2: estimated TCD, mm:  mean (SD) | Repetition #3: estimated TCD, mm:  mean (SD) |
| --- | --- | --- | --- | --- |
| 16 | 15.7 (0.2) | 15.7 (0.1) | 15.7 (0.2) | 15.7 (0.3) |
| 17 | 17.2 (0.1) | 17.2 (0.1) | 17.1 (0.1) | 17.2 (0.1) |
| 18 | 18.7 (0.5) | 18.9 (0.6) | 18.6 (0.4) | 18.5 (0.5) |
| 19 | 21.6 (0.1) | 21.6 (0.1) | 21.6 (0.1) | 21.6 (0.1) |
| 20 | 21 (0.5) | 21.2 (0.5) | 21.1 (0.6) | 20.9 (0.7) |
| 21 | 21.9 (0.2) | 21.9 (0.1) | 22 (0.2) | 22 (0.3) |
| 22 | 22.4 (0.2) | 22.4 (0.2) | 22.4 (0.2) | 22.4 (0.2) |
| 23 | 24.4 (0.2) | 24.4 (0.2) | 24.4 (0.3) | 24.4 (0.2) |
| 24 | 24.5 (0.3) | 24.6 (0.3) | 24.4 (0.2) | 24.5 (0.3) |
| 25 | 28.9 (0.2) | 28.9 (0.2) | 28.9 (0.2) | 28.9 (0.3) |
| 26 | 30.2 (0.5) | 30.8 (0.5) | 30.3 (0.3) | 30.1 (0.3) |
| 27 | 31.3 (0.5) | 31.3 (0.5) | 31.4 (0.4) | 31.5 (0.4) |
| 28 | 33.9 (0.6) | 34 (0.5) | 33.8 (0.6) | 34 (0.6) |
| 29 | 35.6 (1.5) | 35.4 (1) | 35.6 (1.2) | 35.8 (1.9) |
| 30 | 37.5 (1.4) | 37.4 (1.3) | 38 (1) | 36.9 (1.1) |
| 31 | 39.1 (0.5) | 39.4 (0.4) | 39.2 (0.6) | 39.3 (0.5) |
| 32 | 40.2 (1.1) | 40.2 (1) | 40.5 (1.3) | 40.3 (1.4) |
| 33 | 42.9 (1.4) | 43.1 (1.2) | 42.2 (1.7) | 42.9 (1.8) |
| 34 | 44.8 (0.5) | 44.8 (0.1) | 44.8 (0.2) | 44.6 (0.5) |
| 35 | 46.7 (1.5) | 46.2 (1.5) | 47.4 (1.7) | 46.7 (1.1) |
| 36 | 47.8 (3.8) | 46.6 (3.5) | 47.8 (3.2) | 48.7 (1.9) |
| 37 | 50.3 (1.3) | 50.5 (1.9) | 50.3 (0.4) | 50.5 (0.7) |
| 38 | 51.3 (1.3) | 50.9 (1.3) | 51.5 (1.3) | 51.2 (2.2) |
| 39 | 57.2 (0.5) | 57 (0.5) | 57.1 (0.2) | 57.2 (0.4) |
| 40 | 58.1 (2.3) | 60.4 (1.1) | 56.8 (1.2) | 60.8 (0.6) |
