# Supplementary material for: Postnatal gestational age determination by ultrasonographic measurement of transverse cerebellar diameter: A simulation study
Source: PLoS One. 2026 Aug 24;21(8):e0356370. doi: 10.1371/journal.pone.0356370 (PMC13502679; doi:10.1371/journal.pone.0356370)
Supplement: Supplementary file 8 [file pone.0356370.s006.docx]

Table S6. The simulation process was checked using three repetitions for Study #4, which correctly produced consistent simulated data. GA: gestational age. SD: standard deviation. TCD: transverse cerebellar diameter.

| Menstrual GA weeks | Reported TCD, mm: mean (SD) | Repetition #1: estimated TCD, mm:  mean (SD) | Repetition #2: estimated TCD, mm:  mean (SD) | Repetition #3: estimated TCD, mm:  mean (SD) |
| --- | --- | --- | --- | --- |
| 18 | 18.5 (0.4) | 18.6 (0.3) | 18.5 (0.4) | 18.6 (0.4) |
| 19 | 20.7 (0.3) | 20.7 (0.4) | 20.7 (0.3) | 20.6 (0.3) |
| 20 | 21.1 (0.2) | 21.1 (0.2) | 21.2 (0.2) | 21.1 (0.2) |
| 21 | 21.8 (0.2) | 21.7 (0.2) | 21.8 (0.2) | 21.8 (0.2) |
| 22 | 22.6 (0.3) | 22.6 (0.2) | 22.6 (0.3) | 22.5 (0.3) |
| 23 | 24.3 (0.3) | 24.3 (0.3) | 24.4 (0.4) | 24.3 (0.2) |
| 24 | 25.2 (0.4) | 24.9 (0.5) | 25.2 (0.3) | 25.1 (0.4) |
| 25 | 27 (0.3) | 27 (0.2) | 27.1 (0.4) | 27 (0.2) |
| 26 | 29.5 (0.4) | 29.5 (0.5) | 29.5 (0.4) | 29.4 (0.3) |
| 27 | 30.3 (0.4) | 30.4 (0.4) | 30.3 (0.6) | 30.3 (0.4) |
| 28 | 33.4 (0.5) | 33.5 (0.4) | 33.5 (0.5) | 33.4 (0.5) |
| 29 | 34.8 (1.2) | 34.7 (1.1) | 35 (1.4) | 34.9 (1.4) |
| 30 | 35.6 (1.5) | 35.8 (1.5) | 36 (1.5) | 35.4 (1.6) |
| 31 | 37.2 (1.5) | 37.4 (1.2) | 37.5 (1.6) | 36.7 (1.7) |
| 32 | 38.4 (1.6) | 38.1 (1.4) | 38.6 (1.6) | 38.4 (1.7) |
| 33 | 40.2 (1.5) | 39.9 (1.7) | 40.4 (1) | 40.1 (1.4) |
| 34 | 42.4 (1.5) | 42.9 (1.2) | 42.5 (1.1) | 42.3 (1.7) |
| 35 | 43.8 (1.6) | 43.4 (1.6) | 43.4 (1.7) | 43.7 (1.8) |
| 36 | 45.6 (1.8) | 46 (2.2) | 45.5 (1.6) | 45.8 (1.9) |
| 37 | 47.4 (1.8) | 46.6 (1.7) | 48.1 (2.2) | 47.7 (2.3) |
| 38 | 48.5 (1.9) | 48.5 (1.8) | 48.5 (2.4) | 48.5 (2) |
| 39 | 50.2 (2.2) | 51 (1.5) | 50.5 (1.3) | 49.3 (1.9) |
| 40 | 51.5 (2.5) | 50.8 (2.7) | 51.4 (2.2) | 49.7 (2.9) |
