# Supplementary material for: Postnatal gestational age determination by ultrasonographic measurement of transverse cerebellar diameter: A simulation study
Source: PLoS One. 2026 Aug 24;21(8):e0356370. doi: 10.1371/journal.pone.0356370 (PMC13502679; doi:10.1371/journal.pone.0356370)
Supplement: Supplementary file 9 [file pone.0356370.s007.docx]

Table S7. Sensitivity analyses: simulated data of transverse cerebellar diameter were obtained assuming different weekly distributions (Normal, uniform, skewed, heavy tail) and used to estimate gestational age according to the equation by Davies et al. The pooled bias (difference between estimated vs. actual gestational age) and 95% limits of agreement suggested comparable estimates assuming different weekly distribution.

| Simulated distribution | Domain | Pooled bias, days | 95% limits of agreement, days |
| --- | --- | --- | --- |
| Normal | 23-32 weeks | 6.0 | -6.7 to 18.7 |
|  | 33-36 weeks | -1.5 | -16.8 to 13.8 |
|  | 23-36 weeks | 4.2 | -9.8 to 18.1 |
|  | 37-40 weeks | -5.0 | -25.8 to 15.8 |
|  | 23-40 weeks | 2.8 | -13.0 to 18.6 |
| Uniform | 23-32 weeks | 6.1 | -6.4 to 18.8 |
|  | 33-36 weeks | -2.1 | -19.8 to 15.5 |
|  | 23-36 weeks | 4.0 | -16.2 to 19.7 |
|  | 37-40 weeks | -4.4 | -23.3 to 14.5 |
|  | 23-40 weeks | 2.6 | -13.5 to 18.6 |
| Skewed distribution | 23-32 weeks | 6.2 | -6.3 to 18.9 |
|  | 33-36 weeks | -1.5 | -18.7 to 15.7 |
|  | 23-36 weeks | 4.3 | -10.1 to 18.7 |
|  | 37-40 weeks | -3.5 | -26.6 to 19.6 |
|  | 23-40 weeks | 2.9 | -13.4 to 19.1 |
| Heavy tail distribution | 23-32 weeks | 5.9 | -7.1 to 18.9 |
|  | 33-36 weeks | -1.5 | -19.0 to 16.0 |
|  | 23-36 weeks | 4.0 | -10.7 to 18.7 |
|  | 37-40 weeks | -4.5 | -28.5 to 19.5 |
|  | 23-40 weeks | 2.6 | -13.9 to 19.1 |
