# Supplementary material for: Postnatal gestational age determination by ultrasonographic measurement of transverse cerebellar diameter: A simulation study
Source: PLoS One. 2026 Aug 24;21(8):e0356370. doi: 10.1371/journal.pone.0356370 (PMC13502679; doi:10.1371/journal.pone.0356370)
Supplement: Supplementary file 10 [file pone.0356370.s017.docx]

May 28, 2026

Review of the manuscript titled:

**Postnatal Gestational Age Determination by Ultrasonographic Measurement of Transverse Cerebellar Diameter: a Simulation Study**

1*. Strengths of the work*

- Clinically relevant and well-justified problem statement (Introduction, pp. 8–9).

- Transparent use of the published equation and explicit definition of the original domain (pp. 9, 12).

- Clear presentation of aggregated and gestational-age–stratified results; use of appropriate methods (Bland–Altman, Tipton for pooling) (pp. 9, 11–13).

2*. Major weaknesses (to be addressed)*

A**. Exclusive reliance on simulations based on means/SD**

- Risk of oversimplification: the true weekly distributions may be non-normal, heteroscedastic, or skewed. The authors mention this (p. 14), but must quantify the impact.

- Required: more extensive sensitivity analyses.

B. **Selection and quality of source studies**

- Only 4 studies (3 Nigeria, 1 India) — possible lack of geographic and phenotypic representativeness. The authors should discuss how population differences (mean fetal size, growth patterns) could affect the equation, or provide evidence that it is robust to population characteristics and cite it.

- Provide a supplementary table with means/SD by week (if not already complete in the supplement, ensure it can be downloaded and opened) and discuss possible data-extraction errors (pp. 10–11, Table 1).

**C. Justification of the normality assumption and the simulation process**

- The authors should empirically justify weekly normality of TCD (show QQ plots or compare to empirical distributions when possible — include in supplement).

- Number of simulation repetitions and variability between repetitions: specify how many simulations (niter) were used for the main analyses; present uncertainty intervals for the pooled bias that arise from the simulation process itself.

**D. Statistical pooling methods**

- Explain in more detail the method used to calculate pooled bias and pooled LoA (Tipton reference is present, p. 12). State whether a fixed- or random-effects model was used and give the exact formula; provide R code in the supplement.

**E. Clinical interpretation of the LoA**

- The reported LoA (e.g., for 23–32 wks: −6.7 to +18.7 days) are wide — the claim of “acceptable for clinical use” needs qualification. I recommend clearly stating which clinical decisions (p. 13) these limits would be sufficient for (for example: identifying <28 wks vs ≥28 wks). Propose performing classification analyses (sensitivity/specificity) for clinically relevant thresholds (extreme preterm, very preterm, term). To date, clinical utility has not been demonstrated.

3*. Minor weaknesses and editorial corrections*

- Spelling/grammar: several small errors (“Whitin” in abstract p. 1).

- Clarify numbers: on p. 11 there is a figure “7.1 days (95% LoA -1-1 to 15.4 days)”

- Include in Methods the exact simulation parameters (seed, niter) and number of repetitions per dataset (3 repetitions are mentioned as a check; assess whether this is statistically sufficient for inference and justify).

4. *Conclusions*

- The study addresses an interesting contribution but requires:

- greater rigor in presenting and justifying the simulation procedure,

- sensitivity and robustness analyses,

- clearer statistical explanations and reproducible supplementary materials,

- language revision and correction of numerical errors.

Since they mention it in the introduction, the Discussion should address resource-availability issues: they state that prenatal ultrasound may not be available in low- and middle-income countries — the authors should discuss how postnatal ultrasound access could be achieved. Ultimately, this method could serve as an additional tool for assessment, although the ideal remains appropriate prenatal evaluations for accurate dating and growth monitoring. If the authors address the major issues and provide additional analyses and reproducible materials, the manuscript could be accepted.
