# Supplementary material for: Postnatal gestational age determination by ultrasonographic measurement of transverse cerebellar diameter: A simulation study
Source: PLoS One. 2026 Aug 24;21(8):e0356370. doi: 10.1371/journal.pone.0356370 (PMC13502679; doi:10.1371/journal.pone.0356370)
Supplement: Supplementary file 11 [file pone.0356370.s019.docx]

Second review of the manuscript titled:

**Postnatal Gestational Age Determination by Ultrasonographic Measurement of Transverse Cerebellar Diameter: a Simulation Study**

Some of the suggested corrections were implemented, but the manuscript could be further improved by addressing the following points:

- Include a brief PRISMA flow diagram showing why only four studies were included, and explain how the selection does not (or does) limit geographic/phenotypic representativeness and generalizability.
- Provide the TCD measurement technique used in each source study; discuss how differences in technique may bias TCD and therefore estimated GA. Confirm and state whether measurements were prenatal fetal or postnatal, and discuss the impact.
- Specify niter (number of simulation iterations per study) used in the main analyses.
- Provide the random seeds and describe exactly how the “skewed” and “heavy-tail” distributions were generated (parameters), or refer to the supplementary material where this is documented.
- Include in the supplementary material: (a) executable R code that reproduces each figure and table, (b) the method used to estimate τ2 (e.g., DerSimonian–Laird, REML), and (c) heterogeneity metrics (I2 / τ2) and their impact on pooled limits of agreement.
- Expand the Discussion to explicitly acknowledge limitations arising from using algorithm-generated values and the need for clinical validation; state that the true utility of this tool as clinical support will only be known after validation in real clinical settings, and discuss constraints on implementation in different clinical environments (e.g., limited access to ultrasound equipment).
