# Supplementary material for: Postnatal gestational age determination by ultrasonographic measurement of transverse cerebellar diameter: A simulation study
Source: PLoS One. 2026 Aug 24;21(8):e0356370. doi: 10.1371/journal.pone.0356370 (PMC13502679; doi:10.1371/journal.pone.0356370)
Supplement: Supplementary file 12 [file pone.0356370.s020.docx]

Padova, June 25, 2026

Manuscript PONE-D-26-12831

Dear Sikolia Wanyonyi
Academic Editor
PLOS One

Enclosed please find the revised version of our manuscript entitled “Postnatal Gestational Age Determination by Ultrasonographic Measurement of Transverse Cerebellar Diameter: a Simulation Study”.

We thank the Editor and the Reviewers for their comments, and we hope to have satisfactorily answered to all queries.

Best regards,

Andrea Pietravalle,

Doctors with Africa CUAMM, Padua, Italy

**Comments to the Author**

**Reviewer #1**:

1. This is an important research topic for the LMIC. The statistical analysis is clear. The study period is however not stated. Only 4 studies are selected, and it is not clear if these were the only studies available in the study period? There is no exclusion criteria which then makes this an assumption that these were the only studies. In the selected studies which approach was used to measure the TCD - transmastoid vs anterior fontanelle?

**Re:** The four articles were the only studies available in the study period (from database inception to January 2026). We added such information in the revised manuscript: “The search was carried out in January 2026 without time limitations.”. (Methods, lines 103-104). We also specified that “All studies measured TCD during routine ultrasonographic fetal biometry examinations.” (Results, lines 166-167).

2. Line 39: please review is this for beyond 23 weeks or do you mean 32 weeks. It has been stated before that there was benefit 23-32 weeks.

**Re:** We thank the Reviewer for noticing this typo. We change the sentence as “Lower precision, yet acceptable for clinical use, may be expected beyond 32 weeks’ gestation.” (lines 36-37).

3. Line 67: I would suggest that you add the formulae at this stage (22)

**Re:** We added the formula in the Introduction as suggested: “Davies et al. tested TCD measurement by cranial ultrasound for determining GA of preterm infants and defined an equation for prediction of GA (GA in weeks = 0.470 × TCD in mm + 13.162 ) with an accuracy of ± 16.3 days in the original study and ± 13.8 days in a subsequent study. [22,23]” (lines 68-72).

**Reviewer #2**:

1. Strengths of the work
- Clinically relevant and well-justified problem statement (Introduction, pp. 8–9).
- Transparent use of the published equation and explicit definition of the original domain (pp. 9, 12).
- Clear presentation of aggregated and gestational-age–stratified results; use of appropriate methods (Bland–Altman, Tipton for pooling) (pp. 9, 11–13).

**Re:** We thank the Reviewer for the comment.

2. Major weaknesses (to be addressed):

A. Exclusive reliance on simulations based on means/SD
- Risk of oversimplification: the true weekly distributions may be non-normal, heteroscedastic, or skewed. The authors mention this (p. 14), but must quantify the impact.
- Required: more extensive sensitivity analyses.

**Re:** We originally assumed normality of TCD as the original papers presented the data as mean and SD, which implies that the authors checked for normality in their original datasets. However, we acknowledged the risk of oversimplification in the end of the Discussion, as the true distribution may be non-normal. In the revised manuscript, we added a sensitivity analysis assuming non-normal distributions: “The sensitivity analysis suggested comparable GA estimates when different weekly distributions (Normal, uniform, skewed, heavy tail) were used to simulate TCD data. For GA within 23–32 weeks, pooled bias was 6.0 days (95% LoA -6.7 to 18.7 days) with Normal distribution, 6.1 days (95% LoA -6.4 to 18.8 days) with uniform distribution, 6.2 days (95% LoA -6.3 to 18.9 days) with skewed distribution, and 5.9 days (95% LoA -7.1 to 18.9 days) with heavy-tailed distribution. For GA within 23–40 weeks, pooled bias was 2.8 days (95% LoA -13.0 to 18.6 days) with Normal distribution, 2.6 days (95% LoA -13.5 to 18.6 days) with uniform distribution, 2.9 days (95% LoA -13.4 to 19.1 days) with skewed distribution, and 2.6 days (95% LoA -13.9 to 19.1 days) with heavy-tailed distribution. All numerical results are reported in Supplementary Table 6.” (Results, lines 225-236). We also added a description in the Statistical Analysis section: “A sensitivity analysis simulated data of TCD assuming different weekly distributions (uniform, skewed, heavy tail) and used these data to estimate GA according to the equation by Davies et al. [22]. Pooled bias and 95% LoA were calculated for each assumption.” (Results, lines 146-150). Finally, we added a consideration in the Discussion section: “First, simulated data may oversimplify real-world data, hence failing to replicate patient heterogeneity, and the true distribution may be non-Normal. However, the sensitivity analysis suggested comparable GA estimates when different weekly distributions (Normal, uniform, skewed, heavy tail) were used to simulate TCD data.” (lines 302-307).

B. Selection and quality of source studies
- Only 4 studies (3 Nigeria, 1 India) — possible lack of geographic and phenotypic representativeness. The authors should discuss how population differences (mean fetal size, growth patterns) could affect the equation, or provide evidence that it is robust to population characteristics and cite it.

Re: We thank the Reviewer for the comment. In the revised manuscript, we added some considerations in the Discussion section: “Finally, a further limitation concerns the geographic representativeness of the source data, as three studies were conducted in Nigeria and one in India. Population-specific differences in fetal anthropometry and growth patterns could theoretically affect the performance of a gestational-age prediction model when applied across different settings. Indeed, ethnic differences in TCD growth curves have been reported, suggesting that population-specific reference charts may improve accuracy in some settings. [41,42] However, the original postnatal TCD equation was developed in Australia and subsequently evaluated in an independent cohort from Spain, showing comparable performance despite the different geographic and population settings. [22,23] Nevertheless, some degree of population-specific variability cannot be excluded, thus caution is suggested in the interpretation of our findings that may be integrated by future research using individual-level data from geographically different populations. (lines 314-328).

- Provide a supplementary table with means/SD by week (if not already complete in the supplement, ensure it can be downloaded and opened) and discuss possible data-extraction errors (pp. 10–11, Table 1).

**Re:** Supplementary Table 1 displays means/SD by week as were reported in the corresponding articles. There were no data-extraction errors since these data were clearly outlined in tables in the corresponding articles.

C. Justification of the normality assumption and the simulation process
- The authors should empirically justify weekly normality of TCD (show QQ plots or compare to empirical distributions when possible — include in supplement).
**Re:** In our study, we assumed normality of TCD as the original papers presented the data as mean and SD, which implies that the authors checked for normality in their original datasets. Unfortunately, empirical distributions cannot be built from the papers as the raw data were not provided, thus we cannot show quantile-quantile plots or make comparisons with empirical distributions. Of course, checking for normality in the simulated data is pointless as they were generated from a random normal distribution. Nonetheless, we acknowledged the risk of oversimplification in the end of the Discussion, as the true distribution may be non-normal. In the revised manuscript, we added a sensitivity analysis assuming non-normal distributions: “The sensitivity analysis suggested comparable GA estimates when different weekly distributions (Normal, uniform, skewed, heavy tail) were used to simulate TCD data. For GA within 23–32 weeks, pooled bias was 6.0 days (95% LoA -6.7 to 18.7 days) with Normal distribution, 6.1 days (95% LoA -6.4 to 18.8 days) with uniform distribution, 6.2 days (95% LoA -6.3 to 18.9 days) with skewed distribution, and 5.9 days (95% LoA -7.1 to 18.9 days) with heavy-tailed distribution. For GA within 23–40 weeks, pooled bias was 2.8 days (95% LoA -13.0 to 18.6 days) with Normal distribution, 2.6 days (95% LoA -13.5 to 18.6 days) with uniform distribution, 2.9 days (95% LoA -13.4 to 19.1 days) with skewed distribution, and 2.6 days (95% LoA -13.9 to 19.1 days) with heavy-tailed distribution. All numerical results are reported in Supplementary Table 6.” (Results, lines 225-236). We also added a description in the Statistical Analysis section: “A sensitivity analysis simulated data of TCD assuming different weekly distributions (uniform, skewed, heavy tail) and used these data to estimate GA according to the equation by Davies et al. [22]. Pooled bias and 95% LoA were calculated for each assumption.” (Results, lines 146-150). Finally, we added a consideration in the Discussion section: “First, simulated data may oversimplify real-world data, hence failing to replicate patient heterogeneity, and the true distribution may be non-Normal. However, the sensitivity analysis suggested comparable GA estimates when different weekly distributions (Normal, uniform, skewed, heavy tail) were used to simulate TCD data.” (lines 302-307).

- Number of simulation repetitions and variability between repetitions: specify how many simulations (niter) were used for the main analyses; present uncertainty intervals for the pooled bias that arise from the simulation process itself.

**Re:** The simulation process generated one dataset for each selected paper using the summary statistics of TCD that the authors provided for each GA. These data are reported in Supplementary Table 1. Unfortunately, the original raw data were not provided in the papers, hence we could not use the raw data to inform the simulation process (that was based on summary statistics of TCD for each GA) and we cannot quantify the pooled bias that arose from our simulation process. We add this consideration in the revised manuscript: “Third, the quality of the simulation depends on the quality of both assumptions and input data, hence suggesting caution in the interpretation of the results. Unfortunately, original raw data were not provided in the papers, hence we could not use raw data to inform the simulation process or quantify the bias that arose from our simulation process.” (Discussion, lines 309-314). However, we were able to check that the simulation process correctly produced different simulated data in each repetition, as shown in Supplementary Figures 1-4. In addition, we checked that simulated data were consistent with the original data distribution, as shown in Supplementary Tables 2-5. These consideration are provided in Methods section: “The simulation process was checked using three repetitions for each dataset [31]: we aimed to obtain i) different simulated data in the three repetitions, and ii) simulated data consistent with the original data distribution.” (lines 118-121) and in the Results section: “The simulation process was checked using three repetitions for each dataset, which correctly produced different simulated data (Supplementary Figures 1-4) that were consistent with the original data distribution (Supplementary Table 2-5). The simulated datasets used in the analysis are reported as Supplementary File.” (lines 173-177).

D. Statistical pooling methods
- Explain in more detail the method used to calculate pooled bias and pooled LoA (Tipton reference is present, p. 12). State whether a fixed- or random-effects model was used and give the exact formula; provide R code in the supplement.

**Re:** In the revised manuscript, we explained the framework by Tipton et al. with the formulas and specified the use of a random-effects model: “Pooled bias and 95% LoA were calculated according to the framework by Tipton et al. using a random-effects model. [32] Pooled bias was calculated as ∑ w_1j_D_j_/∑ w_1j_ , where D_j_ is the bias in study j and the weights w_1j_ are defined to be inverse-variance. LoA were calculated as D±2√{exp[log(S^2^)]+T^2^}, where S^2^ is the estimated average study-variation and T^2^ is the between-study heterogeneity in bias.” (Methods, lines 130-135). We also uploaded the R code as supplementary material.

E. Clinical interpretation of the LoA
- The reported LoA (e.g., for 23–32 wks: −6.7 to +18.7 days) are wide — the claim of “acceptable for clinical use” needs qualification. I recommend clearly stating which clinical decisions (p. 13) these limits would be sufficient for (for example: identifying <28 wks vs ≥28 wks). Propose performing classification analyses (sensitivity/specificity) for clinically relevant thresholds (extreme preterm, very preterm, term). To date, clinical utility has not been demonstrated.

**Re:** In our opinion, the potential clinical utility of postnatal TCD measurements lies primarily in the identification of infants within clinically relevant gestational-age categories, particularly the most immature newborns. In low-resource settings, where reliable prenatal dating is frequently unavailable and birth weight may be the only available proxy for maturity, an uncertainty of approximately ±2 weeks may still provide clinically useful information. Specifically, it may reduce the risk of failing to identify extremely and very preterm infants who require enhanced monitoring and targeted interventions. In the revised manuscript, we revised the Discussion to further clarify that the proposed approach should be viewed as a tool for broad gestational-age categorization rather than precise postnatal dating: “A disagreement of approximately ±2 weeks can be considered acceptable and may provide clinically useful support for broad gestational-age categorization in low-resource settings where reliable prenatal dating is unavailable. Specifically, it may reduce the risk of failing to identify extremely preterm (<28 gestational weeks) and very preterm (28-31 gestational weeks) infants. Accuracy may be lower for moderate preterm (32-33 gestational weeks) and late preterm (34-36 gestational weeks), but the clinical risk is also progressively reduced for these age groups.” (lines 257-270). We also thank the Reviewer for the suggestion about performing classification analyses for clinically relevant thresholds. In the revised manuscript, we added such results: “Table 2 shows the classification analyses for clinically relevant thresholds (extreme preterm, very preterm, late preterm, term). The positive and negative likelihood ratios suggested that classification based on equation-estimated GA had high discriminatory ability, although the performance decreased from extreme preterm to term thresholds.” (lines 216-220 and Table 2). These findings were remarked in the Discussion section: “Nonetheless, the classification in clinically relevant strata (extreme preterm, very preterm, late preterm, term) based on equation-estimated GA suggested high discriminatory ability, suggesting an overall clinical utility. Of note, the performance decreased from extreme preterm to term strata, which may be due to the original domain of the equation. [22]” (lines 266-270). We also added some information in the Methods section: “In addition, a classification analysis for clinically relevant thresholds (extreme preterm, very preterm, late preterm, term) was performed by calculating positive and negative likelihood ratios using the package mada [32]. These metrics were preferred to sensitivity and specificity because these are interrelated hence pooling can be misleading, and the bivariate approach could not be employed due to the small number of studies [33,34].” (lines 140-146).

3. Minor weaknesses and editorial corrections
- Spelling/grammar: several small errors (“Whitin” in abstract p. 1).
- Clarify numbers: on p. 11 there is a figure “7.1 days (95% LoA -1-1 to 15.4 days)”
- Include in Methods the exact simulation parameters (seed, niter) and number of repetitions per dataset (3 repetitions are mentioned as a check; assess whether this is statistically sufficient for inference and justify).

**Re:** We thank the Reviewer for noticing spelling/grammar and text errors, which were corrected in the revised manuscript. Regarding the simulation process, we generated one dataset for each selected paper using the summary statistics of TCD that the authors provided for each GA. These data are reported in Supplementary Table 1. Unfortunately, the original raw data were not provided in the papers, hence we could not use the raw data to inform the simulation process (that was based only on summary statistics of TCD for each GA). The seed is included in the R code uploaded as Supplementary Material and the number of repetitions per dataset was chosen according to White et al. 2024 (ref #31).

4. Conclusions
- The study addresses an interesting contribution but requires:
- greater rigor in presenting and justifying the simulation procedure,
- sensitivity and robustness analyses,
- clearer statistical explanations and reproducible supplementary materials,
- language revision and correction of numerical errors.
Since they mention it in the introduction, the Discussion should address resource-availability issues: they state that prenatal ultrasound may not be available in low- and middle-income countries — the authors should discuss how postnatal ultrasound access could be achieved. Ultimately, this method could serve as an additional tool for assessment, although the ideal remains appropriate prenatal evaluations for accurate dating and growth monitoring. If the authors address the major issues and provide additional analyses and reproducible materials, the manuscript could be accepted.

**Re:** We thank the Reviewer for the comments that improved the quality of the manuscript and the clarity for the reader. In the revised manuscript, we also added some considerations in the Discussion section: “In resource-limited settings, several barriers hinder access to prenatal care, and obtaining accurate GA dating based on the gold standard (sure LMP or first-trimester ultrasound) is difficult. [36] In fact, antenatal visits take place mostly at Primary Health Care centers, mobile clinics and village health posts, where access to expensive equipment such as an ultrasound scanner and necessary skills are unlikely. On the contrary, the ever-increasing diffusion of portable and reliable ultrasound machines at hospital level and their growing use in neonatal brain ultrasound makes it plausible to use this tool to overcome the difficulty of obtaining accurate GA dating. [25]” (lines 239-248).
