# Supplementary material for: Postnatal gestational age determination by ultrasonographic measurement of transverse cerebellar diameter: A simulation study
Source: PLoS One. 2026 Aug 24;21(8):e0356370. doi: 10.1371/journal.pone.0356370 (PMC13502679; doi:10.1371/journal.pone.0356370)
Supplement: Peer review history

# Peer review: Decision Letter 0

**DOI:** 10.1371/journal.pone.0356370.r001

**Authors:** Sikolia Wanyonyi

15 Jun 2026

Dear Dr. Pietravalle,

Thank you for submitting your manuscript to PLOS ONE. After careful consideration, we feel that it has merit but does not fully meet PLOS ONE’s publication criteria as it currently stands. Therefore, we invite you to submit a revised version of the manuscript that addresses the points raised during the review process.

Please submit your revised manuscript by Jul 30 2026 11:59PM. If you will need more time than this to complete your revisions, please reply to this message or contact the journal office at plosone@plos.org. When you're ready to submit your revision, log on to https://www.editorialmanager.com/pone/ and select the 'Submissions Needing Revision' folder to locate your manuscript file.

- A letter that responds to each point raised by the academic editor and reviewer(s). You should upload this letter as a separate file labeled 'Response to Reviewers'.
- A marked-up copy of your manuscript that highlights changes made to the original version. You should upload this as a separate file labeled 'Revised Manuscript with Track Changes'.
- An unmarked version of your revised paper without tracked changes. You should upload this as a separate file labeled 'Manuscript'.

If you would like to make changes to your financial disclosure, please include your updated statement in your cover letter. Guidelines for resubmitting your figure files are available below the reviewer comments at the end of this letter.

If applicable, we recommend that you deposit your laboratory protocols in protocols.io to enhance the reproducibility of your results. Protocols.io assigns your protocol its own identifier (DOI) so that it can be cited independently in the future. For instructions see: https://journals.plos.org/plosone/s/submission-guidelines#loc-laboratory-protocols. Additionally, PLOS ONE offers an option for publishing peer-reviewed Lab Protocol articles, which describe protocols hosted on protocols.io. Read more information on sharing protocols at https://plos.org/protocols?utm_medium=editorial-email&utm_source=authorletters&utm_campaign=protocols.

As the corresponding author, your ORCID iD is verified in the submission system and will appear in the published article. PLOS supports the use of ORCID, and we encourage all coauthors to register for an ORCID iD and use it as well. Please encourage your coauthors to verify their ORCID iD within the submission system before final acceptance, as unverified ORCID iDs will not appear in the published article. *Only*  the individual author can complete the verification step; PLOS staff *cannot*  verify ORCID iDs on behalf of authors.

We look forward to receiving your revised manuscript.

Kind regards,

Sikolia Wanyonyi

Academic Editor

PLOS One

Journal Requirements:

When submitting your revision, we need you to address these additional requirements.

1. Please ensure that your manuscript meets PLOS ONE's style requirements, including those for file naming. The PLOS ONE style templates can be found at

https://journals.plos.org/plosone/s/file?id=wjVg/PLOSOne_formatting_sample_main_body.pdf and

https://journals.plos.org/plosone/s/file?id=ba62/PLOSOne_formatting_sample_title_authors_affiliations.pdf

2. Please note that PLOS One has specific guidelines on code sharing for submissions in which author-generated code underpins the findings in the manuscript. In these cases, all author-generated code must be made available without restrictions upon publication of the work. Please review our guidelines at https://journals.plos.org/plosone/s/materials-and-software-sharing#loc-sharing-code and ensure that your code is shared in a way that follows best practice and facilitates reproducibility and reuse.

3. If the reviewer comments include a recommendation to cite specific previously published works, please review and evaluate these publications to determine whether they are relevant and should be cited. There is no requirement to cite these works unless the editor has indicated otherwise.

[Note: HTML markup is below. Please do not edit.]

Reviewers' comments:

Reviewer's Responses to Questions

**Comments to the Author**

1. Is the manuscript technically sound, and do the data support the conclusions?

Reviewer #1: Yes

Reviewer #2: Partly

**********

2. Has the statistical analysis been performed appropriately and rigorously?

Reviewer #1: Yes

Reviewer #2: No

**********

3. Have the authors made all data underlying the findings in their manuscript fully available?

The PLOS Data policy

Reviewer #1: Yes

Reviewer #2: Yes

**********

4. Is the manuscript presented in an intelligible fashion and written in standard English?

Reviewer #1: Yes

Reviewer #2: Yes

**********

Reviewer #1: This is an important research topic for the LMIC. The statistical analysis is clear.

The study period is however not stated. Only 4 studies are selected, and it is not clear if these were the only studies available in the study period? There is no exclusion criteria which then makes this an assumption that these were the only studies. In the selected studies which approach was used to measure the TCD - transmastoid vs anterior fontanelle

39: please review is this for beyond 23 weeks or do you mean 32 weeks. It has been stated before that there was benefit 23-32 weeks.

67: I would suggest that you add the formulae at this stage (22)

Reviewer #2: Review of the manuscript titled:

Postnatal Gestational Age Determination by Ultrasonographic Measurement of Transverse Cerebellar Diameter: a Simulation Study

1. Strengths of the work

- Clinically relevant and well-justified problem statement (Introduction, pp. 8–9).

- Transparent use of the published equation and explicit definition of the original domain (pp. 9, 12).

- Clear presentation of aggregated and gestational-age–stratified results; use of appropriate methods (Bland–Altman, Tipton for pooling) (pp. 9, 11–13).

2. Major weaknesses (to be addressed)

A. Exclusive reliance on simulations based on means/SD

- Risk of oversimplification: the true weekly distributions may be non-normal, heteroscedastic, or skewed. The authors mention this (p. 14), but must quantify the impact.

- Required: more extensive sensitivity analyses.

B. Selection and quality of source studies

- Only 4 studies (3 Nigeria, 1 India) — possible lack of geographic and phenotypic representativeness. The authors should discuss how population differences (mean fetal size, growth patterns) could affect the equation, or provide evidence that it is robust to population characteristics and cite it.

- Provide a supplementary table with means/SD by week (if not already complete in the supplement, ensure it can be downloaded and opened) and discuss possible data-extraction errors (pp. 10–11, Table 1).

C. Justification of the normality assumption and the simulation process

- The authors should empirically justify weekly normality of TCD (show QQ plots or compare to empirical distributions when possible — include in supplement).

- Number of simulation repetitions and variability between repetitions: specify how many simulations (niter) were used for the main analyses; present uncertainty intervals for the pooled bias that arise from the simulation process itself.

D. Statistical pooling methods

- Explain in more detail the method used to calculate pooled bias and pooled LoA (Tipton reference is present, p. 12). State whether a fixed- or random-effects model was used and give the exact formula; provide R code in the supplement.

E. Clinical interpretation of the LoA

- The reported LoA (e.g., for 23–32 wks: −6.7 to +18.7 days) are wide — the claim of “acceptable for clinical use” needs qualification. I recommend clearly stating which clinical decisions (p. 13) these limits would be sufficient for (for example: identifying <28 wks vs ≥28 wks). Propose performing classification analyses (sensitivity/specificity) for clinically relevant thresholds (extreme preterm, very preterm, term). To date, clinical utility has not been demonstrated.

3. Minor weaknesses and editorial corrections

- Spelling/grammar: several small errors (“Whitin” in abstract p. 1).

- Clarify numbers: on p. 11 there is a figure “7.1 days (95% LoA -1-1 to 15.4 days)”

- Include in Methods the exact simulation parameters (seed, niter) and number of repetitions per dataset (3 repetitions are mentioned as a check; assess whether this is statistically sufficient for inference and justify).

4. Conclusions

- The study addresses an interesting contribution but requires:

- greater rigor in presenting and justifying the simulation procedure,

- sensitivity and robustness analyses,

- clearer statistical explanations and reproducible supplementary materials,

- language revision and correction of numerical errors.

Since they mention it in the introduction, the Discussion should address resource-availability issues: they state that prenatal ultrasound may not be available in low- and middle-income countries — the authors should discuss how postnatal ultrasound access could be achieved. Ultimately, this method could serve as an additional tool for assessment, although the ideal remains appropriate prenatal evaluations for accurate dating and growth monitoring. If the authors address the major issues and provide additional analyses and reproducible materials, the manuscript could be accepted.

**********

what does this mean?). If published, this will include your full peer review and any attached files.

If you choose “no”, your identity will remain anonymous but your review may still be made public.

**Do you want your identity to be public for this peer review?**  For information about this choice, including consent withdrawal, please see our Privacy Policy

Reviewer #1: No

Reviewer #2: **Yes:** BERENICE VELAZQUEZ TORRES

**********

[NOTE: If reviewer comments were submitted as an attachment file, they will be attached to this email and accessible via the submission site. Please log into your account, locate the manuscript record, and check for the action link "View Attachments". If this link does not appear, there are no attachment files.]

To ensure your figures meet our technical requirements, please review our figure guidelines: https://journals.plos.org/plosone/s/figures

You may also use PLOS’s free figure tool, NAAS, to help you prepare publication quality figures: https://journals.plos.org/plosone/s/figures#loc-tools-for-figure-preparation.

NAAS will assess whether your figures meet our technical requirements by comparing each figure against our figure specifications.

Attachment Submitted filename: posnatal measurement of Transverse Cerebellar Diameter.docx

# Author comment: Author response to Decision Letter 1

**DOI:** 10.1371/journal.pone.0356370.r002

25 Jun 2026

Comments to the Author

Reviewer #1:

1. This is an important research topic for the LMIC. The statistical analysis is clear. The study period is however not stated. Only 4 studies are selected, and it is not clear if these were the only studies available in the study period? There is no exclusion criteria which then makes this an assumption that these were the only studies. In the selected studies which approach was used to measure the TCD - transmastoid vs anterior fontanelle?

Re: The four articles were the only studies available in the study period (from database inception to January 2026). We added such information in the revised manuscript: “The search was carried out in January 2026 without time limitations.”. (Methods, lines 103-104). We also specified that “All studies measured TCD during routine ultrasonographic fetal biometry examinations.” (Results, lines 166-167).

2. Line 39: please review is this for beyond 23 weeks or do you mean 32 weeks. It has been stated before that there was benefit 23-32 weeks.

Re: We thank the Reviewer for noticing this typo. We change the sentence as “Lower precision, yet acceptable for clinical use, may be expected beyond 32 weeks’ gestation.” (lines 36-37).

3. Line 67: I would suggest that you add the formulae at this stage (22)

Re: We added the formula in the Introduction as suggested: “Davies et al. tested TCD measurement by cranial ultrasound for determining GA of preterm infants and defined an equation for prediction of GA (GA in weeks = 0.470 × TCD in mm + 13.162 ) with an accuracy of ± 16.3 days in the original study and ± 13.8 days in a subsequent study. [22,23]” (lines 68-72).

Reviewer #2:

1. Strengths of the work

- Clinically relevant and well-justified problem statement (Introduction, pp. 8–9).

- Transparent use of the published equation and explicit definition of the original domain (pp. 9, 12).

- Clear presentation of aggregated and gestational-age–stratified results; use of appropriate methods (Bland–Altman, Tipton for pooling) (pp. 9, 11–13).

Re: We thank the Reviewer for the comment.

2. Major weaknesses (to be addressed):

A. Exclusive reliance on simulations based on means/SD

- Risk of oversimplification: the true weekly distributions may be non-normal, heteroscedastic, or skewed. The authors mention this (p. 14), but must quantify the impact.

- Required: more extensive sensitivity analyses.

Re: We originally assumed normality of TCD as the original papers presented the data as mean and SD, which implies that the authors checked for normality in their original datasets. However, we acknowledged the risk of oversimplification in the end of the Discussion, as the true distribution may be non-normal. In the revised manuscript, we added a sensitivity analysis assuming non-normal distributions: “The sensitivity analysis suggested comparable GA estimates when different weekly distributions (Normal, uniform, skewed, heavy tail) were used to simulate TCD data. For GA within 23–32 weeks, pooled bias was 6.0 days (95% LoA -6.7 to 18.7 days) with Normal distribution, 6.1 days (95% LoA -6.4 to 18.8 days) with uniform distribution, 6.2 days (95% LoA -6.3 to 18.9 days) with skewed distribution, and 5.9 days (95% LoA -7.1 to 18.9 days) with heavy-tailed distribution. For GA within 23–40 weeks, pooled bias was 2.8 days (95% LoA -13.0 to 18.6 days) with Normal distribution, 2.6 days (95% LoA -13.5 to 18.6 days) with uniform distribution, 2.9 days (95% LoA -13.4 to 19.1 days) with skewed distribution, and 2.6 days (95% LoA -13.9 to 19.1 days) with heavy-tailed distribution. All numerical results are reported in Supplementary Table 6.” (Results, lines 225-236). We also added a description in the Statistical Analysis section: “A sensitivity analysis simulated data of TCD assuming different weekly distributions (uniform, skewed, heavy tail) and used these data to estimate GA according to the equation by Davies et al. [22]. Pooled bias and 95% LoA were calculated for each assumption.” (Results, lines 146-150). Finally, we added a consideration in the Discussion section: “First, simulated data may oversimplify real-world data, hence failing to replicate patient heterogeneity, and the true distribution may be non-Normal. However, the sensitivity analysis suggested comparable GA estimates when different weekly distributions (Normal, uniform, skewed, heavy tail) were used to simulate TCD data.” (lines 302-307).

B. Selection and quality of source studies

- Only 4 studies (3 Nigeria, 1 India) — possible lack of geographic and phenotypic representativeness. The authors should discuss how population differences (mean fetal size, growth patterns) could affect the equation, or provide evidence that it is robust to population characteristics and cite it.

Re: We thank the Reviewer for the comment. In the revised manuscript, we added some considerations in the Discussion section: “Finally, a further limitation concerns the geographic representativeness of the source data, as three studies were conducted in Nigeria and one in India. Population-specific differences in fetal anthropometry and growth patterns could theoretically affect the performance of a gestational-age prediction model when applied across different settings. Indeed, ethnic differences in TCD growth curves have been reported, suggesting that population-specific reference charts may improve accuracy in some settings. [41,42] However, the original postnatal TCD equation was developed in Australia and subsequently evaluated in an independent cohort from Spain, showing comparable performance despite the different geographic and population settings. [22,23] Nevertheless, some degree of population-specific variability cannot be excluded, thus caution is suggested in the interpretation of our findings that may be integrated by future research using individual-level data from geographically different populations. (lines 314-328).

- Provide a supplementary table with means/SD by week (if not already complete in the supplement, ensure it can be downloaded and opened) and discuss possible data-extraction errors (pp. 10–11, Table 1).

Re: Supplementary Table 1 displays means/SD by week as were reported in the corresponding articles. There were no data-extraction errors since these data were clearly outlined in tables in the corresponding articles.

C. Justification of the normality assumption and the simulation process

- The authors should empirically justify weekly normality of TCD (show QQ plots or compare to empirical distributions when possible — include in supplement).

Re: In our study, we assumed normality of TCD as the original papers presented the data as mean and SD, which implies that the authors checked for normality in their original datasets. Unfortunately, empirical distributions cannot be built from the papers as the raw data were not provided, thus we cannot show quantile-quantile plots or make comparisons with empirical distributions. Of course, checking for normality in the simulated data is pointless as they were generated from a random normal distribution. Nonetheless, we acknowledged the risk of oversimplification in the end of the Discussion, as the true distribution may be non-normal. In the revised manuscript, we added a sensitivity analysis assuming non-normal distributions: “The sensitivity analysis suggested comparable GA estimates when different weekly distributions (Normal, uniform, skewed, heavy tail) were used to simulate TCD data. For GA within 23–32 weeks, pooled bias was 6.0 days (95% LoA -6.7 to 18.7 days) with Normal distribution, 6.1 days (95% LoA -6.4 to 18.8 days) with uniform distribution, 6.2 days (95% LoA -6.3 to 18.9 days) with skewed distribution, and 5.9 days (95% LoA -7.1 to 18.9 days) with heavy-tailed distribution. For GA within 23–40 weeks, pooled bias was 2.8 days (95% LoA -13.0 to 18.6 days) with Normal distribution, 2.6 days (95% LoA -13.5 to 18.6 days) with uniform distribution, 2.9 days (95% LoA -13.4 to 19.1 days) with skewed distribution, and 2.6 days (95% LoA -13.9 to 19.1 days) with heavy-tailed distribution. All numerical results are reported in Supplementary Table 6.” (Results, lines 225-236). We also added a description in the Statistical Analysis section: “A sensitivity analysis simulated data of TCD assuming different weekly distributions (uniform, skewed, heavy tail) and used these data to estimate GA according to the equation by Davies et al. [22]. Pooled bias and 95% LoA were calculated for each assumption.” (Results, lines 146-150). Finally, we added a consideration in the Discussion section: “First, simulated data may oversimplify real-world data, hence failing to replicate patient heterogeneity, and the true distribution may be non-Normal. However, the sensitivity analysis suggested comparable GA estimates when different weekly distributions (Normal, uniform, skewed, heavy tail) were used to simulate TCD data.” (lines 302-307).

- Number of simulation repetitions and variability between repetitions: specify how many simulations (niter) were used for the main analyses; present uncertainty intervals for the pooled bias that arise from the simulation process itself.

Re: The simulation process generated one dataset for each selected paper using the summary statistics of TCD that the authors provided for each GA. These data are reported in Supplementary Table 1. Unfortunately, the original raw data were not provided in the papers, hence we could not use the raw data to inform the simulation process (that was based on summary statistics of TCD for each GA) and we cannot quantify the pooled bias that arose from our simulation process. We add this consideration in the revised manuscript: “Third, the quality of the simulation depends on the quality of both assumptions and input data, hence suggesting caution in the interpretation of the results. Unfortunately, original raw data were not provided in the papers, hence we could not use raw data to inform the simulation process or quantify the bias that arose from our simulation process.” (Discussion, lines 309-314). However, we were able to check that the simulation process correctly produced different simulated data in each repetition, as shown in Supplementary Figures 1-4. In addition, we checked that simulated data were consistent with the original data distribution, as shown in Supplementary Tables 2-5. These consideration are provided in Methods section: “The simulation process was checked using three repetitions for each dataset [31]: we aimed to obtain i) different simulated data in the three repetitions, and ii) simulated data consistent with the original data distribution.” (lines 118-121) and in the Results section: “The simulation process was checked using three repetitions for each dataset, which correctly produced different simulated data (Supplementary Figures 1-4) that were consistent with the original data distribution (Supplementary Table 2-5). The simulated datasets used in the analysis are reported as Supplementary File.” (lines 173-177).

D. Statistical pooling methods

- Explain in more detail the method used to calculate pooled bias and pooled LoA (Tipton reference is present, p. 12). State whether a fixed- or random-effects model was used and give the exact formula; provide R code in the supplement.

Re: In the revised manuscript, we explained the framework by Tipton et al. with the formulas and specified the use of a random-effects model: “Pooled bias and 95% LoA were calculated according to the framework by Tipton et al. using a random-effects model. [32] Pooled bias was calculated as ∑ w1jDj/∑ w1j , where Dj is the bias in study j and the weights w1j are defined to be inverse-variance. LoA were calculated as D±2√{exp[log(S2)]+T2}, where S2 is the estimated average study-variation and T2 is the between-study heterogeneity in bias.” (Methods, lines 130-135). We also uploaded the R code as supplementary material.

E. Clinical interpretation of the LoA

- The reported LoA (e.g., for 23–32 wks: −6.7 to +18.7 days) are wide — the claim of “acceptable for clinical use” needs qualification. I recommend clearly stating which clinical decisions (p. 13) these limits would be sufficient for (for example: identifying <28 wks vs ≥28 wks). Propose performing classification analyses (sensitivity/specificity) for clinically relevant thresholds (extreme preterm, very preterm, term). To date, clinical utility has not been demonstrated.

Re: In our opinion, the potential clinical utility of postnatal TCD measurements lies primarily in the identification of infants within clinically relevant gestational-age categories, particularly the most immature newborns. In low-resource settings, where reliable prenatal dating is frequently unavailable and birth weight may be the only available proxy for maturity, an uncertainty of approximately ±2 weeks may still provide clinically useful information. Specifically, it may reduce the risk of failing to identify extremely and very preterm infants who require enhanced monitoring and targeted interventions. In the revised manuscript, we revised the Discussion to further clarify that the proposed approach should be viewed as a tool for broad gestational-age categorization rather than precise postnatal dating: “A disagreement of approximately ±2 weeks can be considered acceptable and may provide clinically useful support for broad gestational-age categorization in low-resource settings where reliable prenatal dating is unavailable. Specifically, it may reduce the risk of failing to identify extremely preterm (<28 gestational weeks) and very preterm (28-31 gestational weeks) infants. Accuracy may be lower for moderate preterm (32-33 gestational weeks) and late preterm (34-36 gestational weeks), but the clinical risk is also progressively reduced for these age groups.” (lines 257-270). We also thank the Reviewer for the suggestion about performing classification analyses for clinically relevant thresholds. In the revised manuscript, we added such results: “Table 2 shows the classification analyses for clinically relevant thresholds (extreme preterm, very preterm, late preterm, term). The positive and negative likelihood ratios suggested that classification based on equation-estimated GA had high discriminatory ability, although the performance decreased from extreme preterm to term thresholds.” (lines 216-220 and Table 2). These findings were remarked in the Discussion section: “Nonetheless, the classification in clinically relevant strata (extreme preterm, very preterm, late preterm, term) based on equation-estimated GA suggested high discriminatory ability, suggesting an overall clinical utility. Of note, the performance decreased from extreme preterm to term strata, which may be due to the original domain of the equation. [22]” (lines 266-270). We also added some information in the Methods section: “In addition, a classification analysis for clinically relevant thresholds (extreme preterm, very preterm, late preterm, term) was performed by calculating positive and negative likelihood ratios using the package mada [32]. These metrics were preferred to sensitivity and specificity because these are interrelated hence pooling can be misleading, and the bivariate approach could not be employed due to the small number of studies [33,34].” (lines 140-146).

3. Minor weaknesses and editorial corrections

- Spelling/grammar: several small errors (“Whitin” in abstract p. 1).

- Clarify numbers: on p. 11 there is a figure “7.1 days (95% LoA -1-1 to 15.4 days)”

- Include in Methods the exact simulation parameters (seed, niter) and number of repetitions per dataset (3 repetitions are mentioned as a check; assess whether this is statistically sufficient for inference and justify).

Re: We thank the Reviewer for noticing spelling/grammar and text errors, which were corrected in the revised manuscript. Regarding the simulation process, we generated one dataset for each selected paper using the summary statistics of TCD that the authors provided for each GA. These data are reported in Supplementary Table 1. Unfortunately, the original raw data were not provided in the papers, hence we could not use the raw data to inform the simulation process (that was based only on summary statistics of TCD for each GA). The see

Attachment Submitted filename: Response to Reviewers.docx

# Peer review: Decision Letter 1

**DOI:** 10.1371/journal.pone.0356370.r003

**Authors:** Sikolia Wanyonyi

13 Jul 2026

Dear Dr. Pietravalle,

Please submit your revised manuscript by Aug 27 2026 11:59PM. If you will need more time than this to complete your revisions, please reply to this message or contact the journal office at plosone@plos.org. When you're ready to submit your revision, log on to https://www.editorialmanager.com/pone/ and select the 'Submissions Needing Revision' folder to locate your manuscript file.

- A letter that responds to each point raised by the academic editor and reviewer(s). You should upload this letter as a separate file labeled 'Response to Reviewers'.
- A marked-up copy of your manuscript that highlights changes made to the original version. You should upload this as a separate file labeled 'Revised Manuscript with Track Changes'.
- An unmarked version of your revised paper without tracked changes. You should upload this as a separate file labeled 'Manuscript'.

If you would like to make changes to your financial disclosure, please include your updated statement in your cover letter. Guidelines for resubmitting your figure files are available below the reviewer comments at the end of this letter.

If applicable, we recommend that you deposit your laboratory protocols in protocols.io to enhance the reproducibility of your results. Protocols.io assigns your protocol its own identifier (DOI) so that it can be cited independently in the future. For instructions see: https://journals.plos.org/plosone/s/submission-guidelines#loc-laboratory-protocols. Additionally, PLOS ONE offers an option for publishing peer-reviewed Lab Protocol articles, which describe protocols hosted on protocols.io. Read more information on sharing protocols at https://plos.org/protocols?utm_medium=editorial-email&utm_source=authorletters&utm_campaign=protocols.

As the corresponding author, your ORCID iD is verified in the submission system and will appear in the published article. PLOS supports the use of ORCID, and we encourage all coauthors to register for an ORCID iD and use it as well. Please encourage your coauthors to verify their ORCID iD within the submission system before final acceptance, as unverified ORCID iDs will not appear in the published article. *Only*  the individual author can complete the verification step; PLOS staff *cannot*  verify ORCID iDs on behalf of authors.

We look forward to receiving your revised manuscript.

Kind regards,

Sikolia Wanyonyi

Academic Editor

PLOS One

Journal Requirements:

If the reviewer comments include a recommendation to cite specific previously published works, please review and evaluate these publications to determine whether they are relevant and should be cited. There is no requirement to cite these works unless the editor has indicated otherwise.

Please review your reference list to ensure that it is complete and correct. If you have cited papers that have been retracted, please include the rationale for doing so in the manuscript text, or remove these references and replace them with relevant current references. Any changes to the reference list should be mentioned in the rebuttal letter that accompanies your revised manuscript. If you need to cite a retracted article, indicate the article’s retracted status in the References list and also include a citation and full reference for the retraction notice.

[Note: HTML markup is below. Please do not edit.]

Reviewers' comments:

Reviewer's Responses to Questions

**Comments to the Author**

Reviewer #2: (No Response)

**********

2. Is the manuscript technically sound, and do the data support the conclusions?

Reviewer #2: Yes

**********

3. Has the statistical analysis been performed appropriately and rigorously?

Reviewer #2: Yes

**********

4. Have the authors made all data underlying the findings in their manuscript fully available?

The PLOS Data policy

Reviewer #2: (No Response)

**********

5. Is the manuscript presented in an intelligible fashion and written in standard English?

Reviewer #2: Yes

**********

Reviewer #2: Second review of the manuscript titled:

Postnatal Gestational Age Determination by Ultrasonographic Measurement of Transverse Cerebellar Diameter: a Simulation Study

Some of the suggested corrections were implemented, but the manuscript could be further improved by addressing the following points:

• Include a brief PRISMA flow diagram showing why only four studies were included, and explain how the selection does not (or does) limit geographic/phenotypic representativeness and generalizability.

• Provide the TCD measurement technique used in each source study; discuss how differences in technique may bias TCD and therefore estimated GA. Confirm and state whether measurements were prenatal fetal or postnatal, and discuss the impact.

• Specify niter (number of simulation iterations per study) used in the main analyses.

• Provide the random seeds and describe exactly how the “skewed” and “heavy-tail” distributions were generated (parameters), or refer to the supplementary material where this is documented.

• Include in the supplementary material: (a) executable R code that reproduces each figure and table, (b) the method used to estimate τ2 (e.g., DerSimonian–Laird, REML), and (c) heterogeneity metrics (I2 / τ2) and their impact on pooled limits of agreement.

• Expand the Discussion to explicitly acknowledge limitations arising from using algorithm-generated values and the need for clinical validation; state that the true utility of this tool as clinical support will only be known after validation in real clinical settings, and discuss constraints on implementation in different clinical environments (e.g., limited access to ultrasound equipment).

**********

what does this mean?). If published, this will include your full peer review and any attached files.

If you choose “no”, your identity will remain anonymous but your review may still be made public.

**Do you want your identity to be public for this peer review?**  For information about this choice, including consent withdrawal, please see our Privacy Policy

Reviewer #2: **Yes:** BERENICE VELAZQUEZ TORRES

**********

[NOTE: If reviewer comments were submitted as an attachment file, they will be attached to this email and accessible via the submission site. Please log into your account, locate the manuscript record, and check for the action link "View Attachments". If this link does not appear, there are no attachment files.]

To ensure your figures meet our technical requirements, please review our figure guidelines: https://journals.plos.org/plosone/s/figures

You may also use PLOS’s free figure tool, NAAS, to help you prepare publication quality figures: https://journals.plos.org/plosone/s/figures#loc-tools-for-figure-preparation.

NAAS will assess whether your figures meet our technical requirements by comparing each figure against our figure specifications.

Attachment Submitted filename: Second review of the manuscript titled.docx

# Author comment: Author response to Decision Letter 2

**DOI:** 10.1371/journal.pone.0356370.r004

29 Jul 2026

Comments to the Author

Reviewer #2:

Some of the suggested corrections were implemented, but the manuscript could be further improved by addressing the following points:

1. Include a brief PRISMA flow diagram showing why only four studies were included, and explain how the selection does not (or does) limit geographic/phenotypic representativeness and generalizability.

Re: We thank the Reviewer for the suggestion and we added the PRISMA flow diagram as Supplementary Figure 1: “Four studies met the inclusion criteria and were used for the simulation process (Supplementary Figure 1). [26-29]” (Results, page 4). In the revised manuscript, we added some considerations about the inclusion criteria limiting the generalizability of the findings to LMICs, and the literature output limiting geographic and phenotypic representativeness: “Overall, the generalizability of the findings may be limited to LMICs due to the inclusion criteria, while geographic and phenotypic representativeness was limited by the literature output that yielded only three studies from Nigeria and one study from India. Population-specific differences in fetal anthropometry and growth patterns could theoretically affect the performance of a gestational-age prediction model when applied across different settings. Indeed, ethnic differences in TCD growth curves have been reported, suggesting that population-specific reference charts may improve accuracy in some settings. [41,42] However, the original postnatal TCD equation was developed in Australia and subsequently evaluated in an independent cohort from Spain, showing comparable performance despite the different geographic and population settings. [22,23] Nevertheless, some degree of population-specific variability cannot be excluded, thus caution is suggested in the interpretation of our findings that may be integrated by future research using individual-level data from geographically different populations.” (Discussion, page 10).

2. Provide the TCD measurement technique used in each source study; discuss how differences in technique may bias TCD and therefore estimated GA. Confirm and state whether measurements were prenatal fetal or postnatal, and discuss the impact.

Re: We thank the Reviewer for the suggestion. We added the TCD measurement technique used in each source study as Supplementary Table 2, with reference in the text of the manuscript: “All four studies employed a basic standard methodological approach to evaluate the TCD during routine prenatal fetal ultrasound (Supplementary Table 2).” (Results, page 4). We added some considerations about differences in technique: “Moreover, we cannot exclude some heterogeneity in TCD measurement technique across the studies, despite the employment of a basic standard methodological approach to evaluate the TCD during routine prenatal fetal ultrasound. [26-29] Such heterogeneity may be due to minor differences in image acquisition, caliper placement, transducer orientation and operator expertise. Since Davies’s equation estimates GA using TCD measures [22], these methodological differences may proportionally translate into slightly different GA estimates and contribute to between-study heterogeneity, which is accounted for by the random-effects pooling of the bias.” (Discussion, pages 10-11). We also added some considerations about prenatal and postnatal measurements: “It should be underlined that Davies’s equation was originally developed using postnatal ultrasound, while all source measurements were prenatal fetal measurements. [22,26-29] The equation aimed to refine the use of TCD as reliable predictor of GA, and recent evidence demonstrated strong agreement between prenatal and postnatal TCD measurements [24], but prospective validation using real postnatal measurements remains necessary.” (Discussion, page 9).

3. Methods: a) Specify niter (number of simulation iterations per study) used in the main analyses.; b) Provide the random seeds and describe exactly how the “skewed” and “heavy-tail” distributions were generated (parameters), or refer to the supplementary material where this is documented; c) Include in the supplementary material: i) executable R code that reproduces each figure and table, (ii) the method used to estimate τ2 (e.g., DerSimonian–Laird, REML), and (iii) heterogeneity metrics (I2 / τ2) and their impact on pooled limits of agreement.

Re: We reported this information (niter, seed, distributions) in the “Supplementary Material - R code” file and added a reference to the supplementary material in the revised manuscript. The code describes how the skewed distribution and heavy-tail distribution were generated and their parameters. In the “Supplementary Material - R code", we specified that “The framework used the method-of-moments estimator T2 for the between-study heterogeneity in bias τ2 as described by the original authors (Tipton et al.).”. In the revised manuscript, we reported the heterogeneity metrics τ2 that are produced by the functions of the framework by Tipton et al. (Results, pages 5-6) and we commented about their impact on the findings: “Our simulation-based evaluation suggested that such equation may provide some benefits in estimating GA between 23-32 weeks, which was the original domain of the equation. [22] When the equation was extended beyond the original domain, the between-study heterogeneity increased and the agreement slightly impaired as could be expected. These findings are correlated as higher between-study heterogeneity produces larger agreement intervals [32], and suggests caution in using the equation beyond the original domain.” (Discussion, page 8).

4. Expand the Discussion to explicitly acknowledge limitations arising from using algorithm-generated values and the need for clinical validation; state that the true utility of this tool as clinical support will only be known after validation in real clinical settings, and discuss constraints on implementation in different clinical environments (e.g., limited access to ultrasound equipment).

Re: We thank the Reviewer for the suggestion. In the revised manuscript, we added some considerations about the use of algorithm-generated values: “The main limitation of our study is the use of algorithm-generated values to assess the performance of Davies’s equation in estimating GA using fetal TCD measurements. [22] Simulated data may oversimplify real-world data, hence failing to replicate patient heterogeneity. In this perspective, our findings provide promising information on the potential application of the equation, but clinical validation remains a paramount to assess its utility in the real world. In addition, the true distribution of TCD may be non-Normal, but the sensitivity analysis suggested comparable GA estimates when different weekly distributions (Normal, uniform, skewed, heavy tail) were used to simulate TCD data. Finally, the quality of the simulation depends on the quality of both assumptions and input data, hence suggesting caution in the interpretation of the results. Unfortunately, original raw data were not provided in the papers, hence we could not use raw data to inform the simulation process or quantify the bias that arose from our simulation process.” (Discussion, page 10). We also added some considerations at the end of the Discussion section: “Due to the key limitation of using simulated data (although derived from actual fetal measurements), the true utility of this tool as clinical support will only be known after validation in real clinical settings and future studies may validate the equation by Davies et al. [22] using TCD measurements from real newborns in low-resource settings where precise GA dating is available. The inherent condition for real-world implementation is clearly the availability of an ultrasound. Despite recent developments of such technology have led to affordable devices and progressive spread in the diagnostics process, local constraints such as limited access to ultrasound equipment and limited expertise can impair the implementation of Davies’s equation in the clinical environments of some LMICs.” (Discussion, page 11).

Attachment Submitted filename: Response to Reviewers Rev2.pdf

# Editor report: Decision Letter 2

**DOI:** 10.1371/journal.pone.0356370.r005

**Authors:** Sikolia Wanyonyi

3 Aug 2026

Postnatal Gestational Age Determination by Ultrasonographic Measurement of Transverse Cerebellar Diameter: a Simulation Study

PONE-D-26-12831R2

Dear Dr. Pietravalle,

We’re pleased to inform you that your manuscript has been judged scientifically suitable for publication and will be formally accepted for publication once it meets all outstanding technical requirements.

Within one week, you’ll receive an e-mail detailing the required amendments. When these have been addressed, you’ll receive a formal acceptance letter and your manuscript will be scheduled for publication.

An invoice will be generated when your article is formally accepted. Please note, if your institution has a publishing partnership with PLOS and your article meets the relevant criteria, all or part of your publication costs will be covered. Please make sure your user information is up-to-date by logging into Editorial Manager at Editorial Manager® and clicking the ‘Update My Information' link at the top of the page. For questions related to billing, please contact billing support.

If your institution or institutions have a press office, please notify them about your upcoming paper to help maximize its impact. If they’ll be preparing press materials, please inform our press team as soon as possible -- no later than 48 hours after receiving the formal acceptance. Your manuscript will remain under strict press embargo until 2 pm Eastern Time on the date of publication. For more information, please contact onepress@plos.org.

Kind regards,

Sikolia Wanyonyi

Academic Editor

PLOS One

# Editor report: Acceptance letter

**DOI:** 10.1371/journal.pone.0356370.r006

**Authors:** Sikolia Wanyonyi

PONE-D-26-12831R2

PLOS One

Dear Dr. Pietravalle,

I'm pleased to inform you that your manuscript has been deemed suitable for publication in PLOS One. Congratulations! Your manuscript is now being handed over to our production team.

At this stage, our production department will prepare your paper for publication. This includes ensuring the following:

* All references, tables, and figures are properly cited

* All relevant supporting information is included in the manuscript submission,

* There are no issues that prevent the paper from being properly typeset

You will receive further instructions from the production team, including instructions on how to review your proof when it is ready. Please keep in mind that we are working through a large volume of accepted articles, so please give us a few days to review your paper and let you know the next and final steps.

Lastly, if your institution or institutions have a press office, please let them know about your upcoming paper now to help maximize its impact. If they'll be preparing press materials, please inform our press team within the next 48 hours. Your manuscript will remain under strict press embargo until 2 pm Eastern Time on the date of publication. For more information, please contact onepress@plos.org.

You will receive an invoice from PLOS for your publication fee after your manuscript has reached the completed accept phase. If you receive an email requesting payment before acceptance or for any other service, this may be a phishing scheme. Learn how to identify phishing emails and protect your accounts at https://explore.plos.org/phishing.

If we can help with anything else, please email us at customercare@plos.org.

Thank you for submitting your work to PLOS One and supporting open access.

Kind regards,

PLOS ONE Editorial Office Staff

on behalf of

Dr. Sikolia Wanyonyi

Academic Editor

PLOS One
